# Global, regional, and national burdens of lower extremity peripheral arterial disease from 1990 to 2021 and projections to 2050: global burden of disease study 2021

**DOI:** 10.3389/fcvm.2025.1592322

**Published:** 2025-10-07

**Authors:** Gaojing Zhang, Xincan Liu, Jianchao Li, Yu Zhao, Zhiyu Yuan, Yun Chen, Shunkai Zhang, Mengxin Chang, Lili Jin, Chunjing Tao, Rongxin Tang, Zhenzhen Lan

**Affiliations:** ^1^ Department of Cardiovascular Diseases, The First Affiliated Hospital of Henan University of Traditional Chinese Medicine, Zhengzhou, Henan, China; ^2^ Collaborative Innovation Center of Prevention and Treatment of Major Diseases by Chinese and Western Medicine, Zhengzhou, Henan, China; ^3^ School of Engineering Medicine, Beihang University, Beijing, China; ^4^ Beijing Advanced Innovation Center for Biomedical Engineering, Beihang University, Beijing, China

**Keywords:** lower extremity peripheral artery disease, global burden of disease, social demographic index, risk factor analysis, join-point

## Abstract

**Background:**

The global and regional burden of lower extremity peripheral artery disease (LEPAD) and its trends have not been systematically studied. Utilizing data from the Global Burden of Diseases, Injuries, and Risk Factors Study (GBD) 2021, this study analyses the global burden and associated risk factors for LEPAD from 1990 to 2021 and predicts its incidence trends to 2050.

**Methods:**

LEPAD-related data including the number of morbidity, mortality, disability-adjusted life years (DALYs), age-standardized rate (ASR), were extracted from the GBD 2021 database. The analysis assesses the burden stratified by social demographic index (SDI), age, and sex. Bayesian age-period-cohort (BAPC) models were used to predict the future age-standardized incidence.

**Findings:**

The global incidence, death, and DALY of LEPAD increased significantly between 1990 and 2021; however, age-standardized incidence rate (ASIR), age-standardized rates of death (ASMR), and age-standardized disability-adjusted life years (ASDR) have shown an overall decline. In addition, ASIR and SDI were positively correlated. Age-specific analyses revealed that ASMR increased with age. The predictions from the BAPC model indicate a slight increase in ASIR over the next 29 years. While high fasting glucose dominated LEPAD DALYs, summary exposure value (SEV) metrics exposed high Low-Density Lipoprotein Cholesterol (LDL-C) as the primary metabolic exposure burden, highlighting a critical prevention gap.

**Interpretation:**

The burden of LEPAD increases progressively with age, and its prevalence is influenced mainly by the SDI. Despite the increased incidence of LEPAD in women, mortality and DALYs were substantially higher in men. The global burden of LEPAD is projected to increase progressively by 2050, representing a major health concern.

## Introduction

Lower extremity peripheral arterial disease (LEPAD) is a condition characterized by reduced blood circulation due to narrowing or occlusion of blood vessels in the lower extremities, primarily caused by atherosclerosis. Clinically, LEPAD manifests as intermittent claudication, resting pain, and abnormalities in skin temperature, color, and sensation (1). LEPAD affects an estimated 236 million people worldwide (2–4) and can lead to serious complications such as amputation and acute lower limb ischemia, severely affecting functional status and quality of life. The 2024 ACC/AHA/AACVPR/APMA/ABC/SCAI/SVM/SVN/SVS/SIR/VESS guidelines for the management of LEPAD (1) highlighted the clinical importance of LEPAD. The 2021 update of the Global Burden of Disease (GBD) (5) recognized LEPAD as an independent disease in cardiovascular diseases, highlighting its global health impact. Therefore, assessing the LEPAD burden is crucial for improving global healthcare delivery; however, a comprehensive assessment of the disease burden of LEPAD is lacking.

This study analyzed the incidence, mortality, and disability-adjusted life years (DALYs) of LEPAD at the global, regional, and national levels from 1990 to 2021, based on the most recent data from the GBD 2021 study. This study mainly focused on age and sex distributions, associated risk factors, and changes in temporal trends, with projections extending to 2050. Our findings may help clinicians, epidemiologists, and health policymakers optimize the allocation of medical resources and develop more targeted public health strategies.

## Methods

### Data collection

The GBD Study 2021 (https://vizhub.healthdata.org/gbd-results/) provides a comprehensive assessment of 371 diseases and injuries and 88 risk factors across 204 countries and territories via the latest epidemiological data and advanced standardized methodologies. The GBD database employs sophisticated methods to address missing data and adjust for confounders. Detailed information on the study design and methodology of the GBD study has been extensively described in the existing GBD literature (6, 7). Information on LEPAD for global populations of different ages, including the corresponding values and 95% uncertainty intervals (UIs) of incidence, mortality, and DALYs, was extracted from the GBD 2021 study data.

### Social demographic index (SDI)

The SDI is a composite indicator developed by the Institute for Health Metrics and Evaluation in 2015 to assess the level of development in a country or region, emphasizing the association between social development and population health outcomes (8). Each country or region has an SDI value ranging from 0 to 1, with higher values indicating better development. In the GBD 2021, 204 countries and regions were categorized into five SDI regions: low, medium-low, medium, medium-high, and high (6).

### Risk factors

In addition to the primary indicators of morbidity, mortality, and DALYs, this study describes the impact of specific risk factors on LEPAD burden through dual complementary approaches. The first is DALYs-based attribution using GBD Compare 2021, which quantifies the health loss attributable to each risk; The second is summary exposure value (SEV) analysis, which independently assesses the population exposure level to risks (standardized to 0%–100%).

### Descriptive analysis

Descriptive analysis was used to describe the burden of LEPAD on the global population. To account for differences in age distribution and temporal trends, age-standardized rates (ASRs) were calculated based on the world-standardized population from the GBD database, and heterogeneity was estimated using 95% UI (9). This study compared the age-standardized incidence rates (ASIRs), age-standardized rates of deaths (ASMRs), and age-standardized disability-adjusted life years (ASDRs) of LEPAD in different age groups, sexes, regions, and countries. The R Studio (version 4.4.0; R Core Team, Vienna, Austria) software was used for data collection and graphing.

### Connected-point regression analysis

We assessed ASR trends between 1990 and 2021 by connecting-point regression model analysis by fitting regressions to the natural logarithms of the ASIR and ASMR over different time periods and then calculating the annual percent change (APC) and corresponding 95% confidence interval (CI) for each period via the average annual percent change (AAPC). The corresponding rates were classified as trending upward or downward if the APC and AAPC estimates and their 95% CIs were consistently above or below zero (10). *P* values less than 0.05 indicates statistical significance.

### Bayesian age-period-cohort (BAPC) modeling

BAPC modeling is a sophisticated statistical tool that combines *a priori* information about unknown parameters with sample information to estimate posterior distributions and infer these unknown parameters more accurately for predicting disease burden (11). Therefore, we used the BAPC and INLA packages in R software to predict the global ASIR burden of LEPAD from 2022 to 2050.

## Results

### Global and regional trends

**Figure 1:**
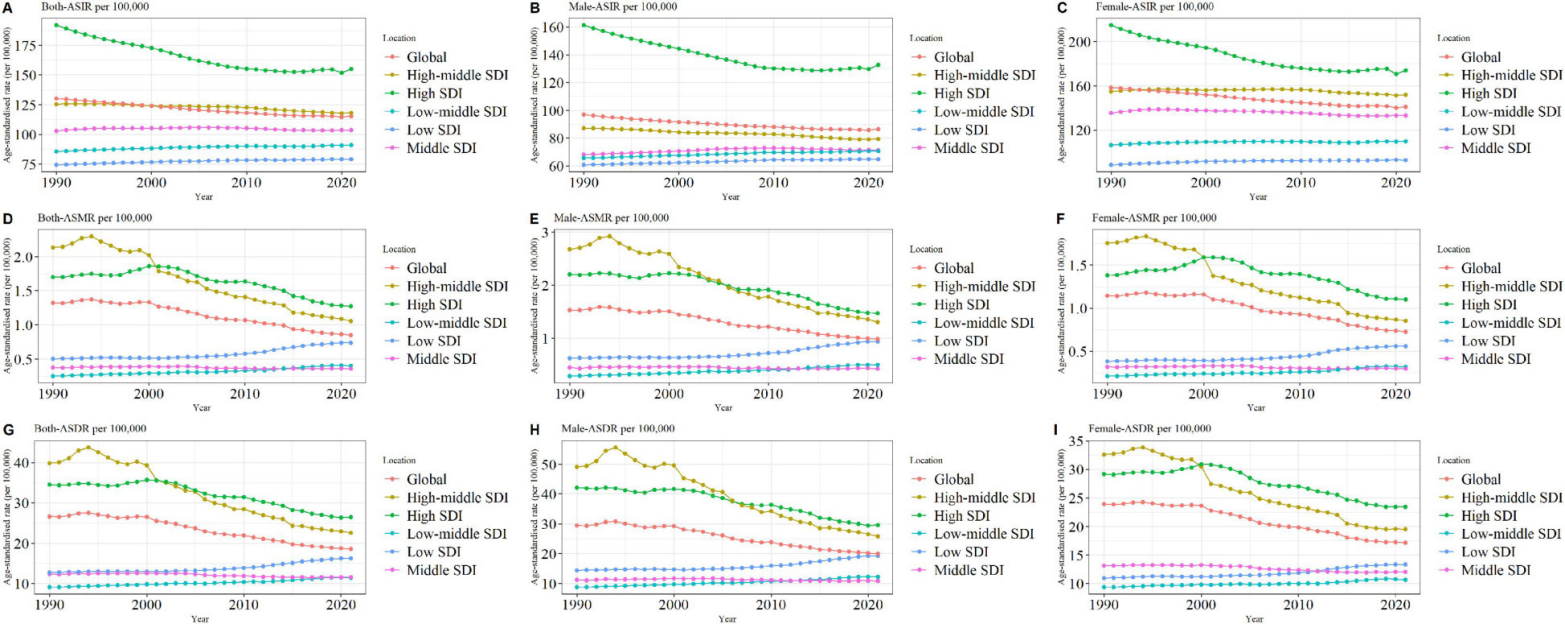
Trends in LEPAD ASIR, ASMR, and ASDR between 1990 and 2021 **(A)** global total population ASIR; **(B)** global ASIR (men); **(C)** global ASIR (women); **(D)** global total population ASMR; **(E)** global ASMR (men); **(F)** global ASMR (women); **(G)** global total population ASDR; **(H)** global ASDR (men); **(I)** global ASDR (women). LEPAD, lower extremity peripheral arterial disease; ASIR, age-standardized incidence rate; ASMR, age-standardized mortality rate; ASDR, age-standardized disability-adjusted life years.

The global burden of LEPAD remained high in 2021, with a substantial increase in morbidity from 50,99,359 in 1990 to 100,38,811 in 2021, an increase in mortality from 39,205 in 1990 to 67,744 in 2021, and the number of DALYs attributable to LEPAD increased by 70.68% from 9,12,987 to 15,58,243 in 2021 (Table 1). The global ASIR, ASMR, and ASDR of LEPAD were lower than those in 1990 (Figure 1). The ASIR in 2021 was 115.44/100,000 [95% (UI) 100.04, 132.72] (Figure 1A), reflecting an 11% decrease from 1990. In particular, the ASIR in women was always higher than that in men (Figures 1B,C). The ASMR decreased from 1.32/100,000 in 1990 [95% (UI) 1.18, 1.43] to 0.85/100,000 [95% (UI) 0.75, 0.93] in 2021 (Figure 1D), with higher ASMR in men than in women (Figures 1E,F). ASDR decreased from 26.62/100,000 [95% (UI) 22.25, 33.81] in 1990 to 18.6/100,000 [95% (UI) 15.18, 24.23] (Figure 1G). Clearly, the ASDR were higher in men than in women (Figures 1H,I).

**Table 1:** Age-standardized incidence rates, age-standardized mortality rates, and age-standardized disability-adjusted life years for lower extremity peripheral arterial disease at the global and regional levels (1990–2021).

Category	Incidence				Deaths				DALYs			
	No. of Cases, 1990 (95% UI)	ASR per 100,000, 1990 (95% UI)	No. of Cases, 2021 (95% UI)	ASR per 100,000, 2021 (95% UI)	No. of Cases, 1990 (95% UI)	ASR per 100,000, 1990 (95% UI)	No. of Cases, 2021 (95% UI)	ASR per 100,000, 2021 (95% UI)	No. of Cases, 1990 (95% UI)	ASR per 100,000, 1990 (95% UI)	No. of Cases, 2021 (95% UI)	ASR per 100,000, 2021 (95% UI)
Global	50,99,359 (44,05,261, 59,11,259)	130.33 (112.75, 149.44)	10,03,8811 (86,89,418, 11,60,1744)	115.44 (100.04, 132.72)	39,205 (35,701, 42,563)	1.32 (1.18, 1.43)	67,744 (59,937, 74,257)	0.85 (0.75, 0.93)	912,987 (754,644, 11,80,311)	26.62 (22.25, 33.81)	15,58,243 (12,66,995, 20,45,869)	18.6 (15.18, 24.23)
Sex												
Male	17,38,552 (15,02,096, 20,19,783)	96.95 (83.956, 111.42)	35,30,125 (30,68,362, 40,88,841)	86.55 (75.35, 99.65)	19,299 (17,751, 21,144)	1.53 (1.41, 1.68)	33,296 (30,493, 37,758)	0.98 (0.9, 1.11)	447,358 (384,434, 532,629)	29.46 (25.49, 34.62)	751,542 (649,221, 922,797)	20.03 (17.26, 24.22)
Female	33,60,807 (29,02,099, 38,81,236)	158.44 (137.55, 182.07)	65,08,687 (56,29,277, 74,76,433)	141.17 (121.95, 162.22)	19,905 (17,569, 21,690)	1.15 (1.00,1.25)	34,447 (29,066, 38,595)	0.73 (0.61, 0.81)	465,629 (365,980, 645,555)	23.95 (19.08, 32.34)	806,701 (610,710, 11,41,010)	17.19 (13.01, 24.31)
Region												
Low SDI	160,019 (137,359, 189,021)	74.31 (64.01, 86.29)	390,151 (335,802, 457,454)	79.12 (67.95, 91.73)	751 (338, 1,465)	0.51 (0.22, 1.00)	2,537 (1,378, 4,814)	0.74 (0.4,1.38)	23,192 (11,909, 39,841)	12.73 (6.59, 21.46)	67,139 (38,284, 111,935)	16.24 (9.31, 26.93)
Low-middle SDI	509,001 (438,679, 597,988)	85.77 (73.94, 99.92)	13,01,037 (11,21,543, 15,28,900)	91.07 (78.47, 105.67)	1,039 (691, 1,555)	0.25 (0.17, 0.38)	4,447 (3,466, 5,724)	0.4 (0.31, 0.52)	45,471 (28,340, 71,711)	9.10 (5.69, 14.01)	144,646 (101,184, 210,747)	11.42 (7.99, 16.4)
Middle SDI	10,58,456 (913,811, 12,37,239)	102.94 (88.97, 119.35)	28,46,599 (24,55,382, 33,27,288)	103.64 (89.39, 120.13)	2,505 (2,251, 2,735)	0.38 (0.33, 0.41)	7,950 (7,078, 8,721)	0.36 (0.32, 0.39)	104,508 (73,544, 157,794)	12.31 (8.78, 18.41)	288,674 (208,505, 425,370)	11.52 (8.33, 16.85)
High-middle SDI	12,39,290 (10,64,965, 14,41,983)	125.41 (108.31, 144.13)	23,58,855 (20,30,754, 27,39,528)	118.33 (102.24, 136.91)	16,431 (14,912, 18,134)	2.13 (1.91, 2.34)	20,196 (18,132, 21,791)	1.06 (0.94,1.14)	352,898 (303,088, 422,183)	39.86 (34.19, 47.06)	445,777 (378,484, 569,422)	22.53 (19.12, 28.57)
High SDI	21,27,669 (18,37,741, 24,30,887)	192.04 (166.78, 220.10)	31,34,025 (27,56,771, 35,41,206)	155.03 (135.92, 176)	18,401 (16,711, 19,481)	1.70 (1.53, 1.81)	32,463 (27,854, 35,213)	1.27 (1.11, 1.38)	3,85,296 (3,27,164, 4,85,072)	34.58 (29.43, 43.22)	6,09,281 (5,12,999, 7,55,404)	26.41 (22.45, 32.86)

DALYs, disability-adjusted life years; No., number; ASR, age-standardised rate; SDI, social demographic index; UI, uncertainty interval.

Significant regional differences were observed in the global burden of LEPAD, which is strongly associated with the SDI (Figure 1), with the most significant differences observed in the ASIR (Figure 1A). In 2021, ASIR was highest in high SDI regions at 155.03 per 100,000 [95% (UI) 135.92, 176] (Figure 1A) and lowest in low SDI regions at 79.12 per 100,000 [95% (UI) 67.95, 91.73] (Figure 1A). Furthermore, the ASIR and SDI were positively correlated in both sexes, indicating that LEPAD prevalence increased with increasing SDI (Figures 1B,C). Interestingly, ASIR decreased in both the high and medium-high SDI regions between 1990 and 2021, with the most significant decrease in the high SDI region whereas ASIR in the low, medium-low, and medium SDI regions showed an increasing trend. As shown in Table 1, the ASIR in the high SDI region decreased from 192.04/100,000 [95% (UI) 166.78, 220.1] in 1990 to 155.03/100,000 [95% (UI) 135.92, 176] in 2021. In contrast, ASIR in the low and medium SDI regions decreased from 85.77/100,000 in 1990 [95% (UI) 73.94, 99.92] to 91.07/100,000 in 2021 [95% (UI) 78.47, 105.67].

### National trends

**Figure 2:**
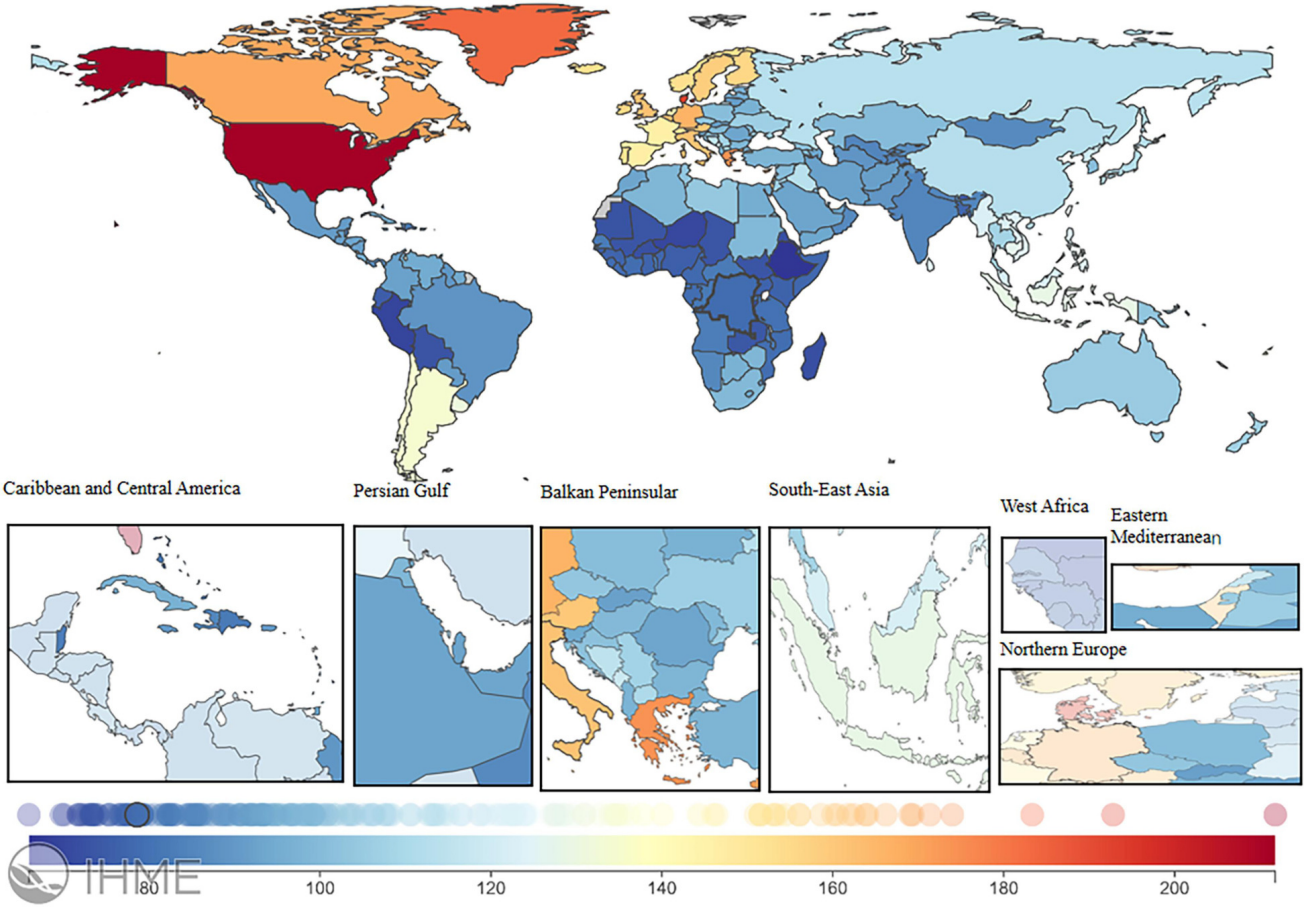
ASIR burden of LEPAD in 204 countries and regions in 2021. LEPAD, lower extremity peripheral artery disease; ASIR, age-standardized incidence rate. Different colors represent different values, from blue to red, indicating a progressive increase in the ASIR burden.

**Figure 3:**
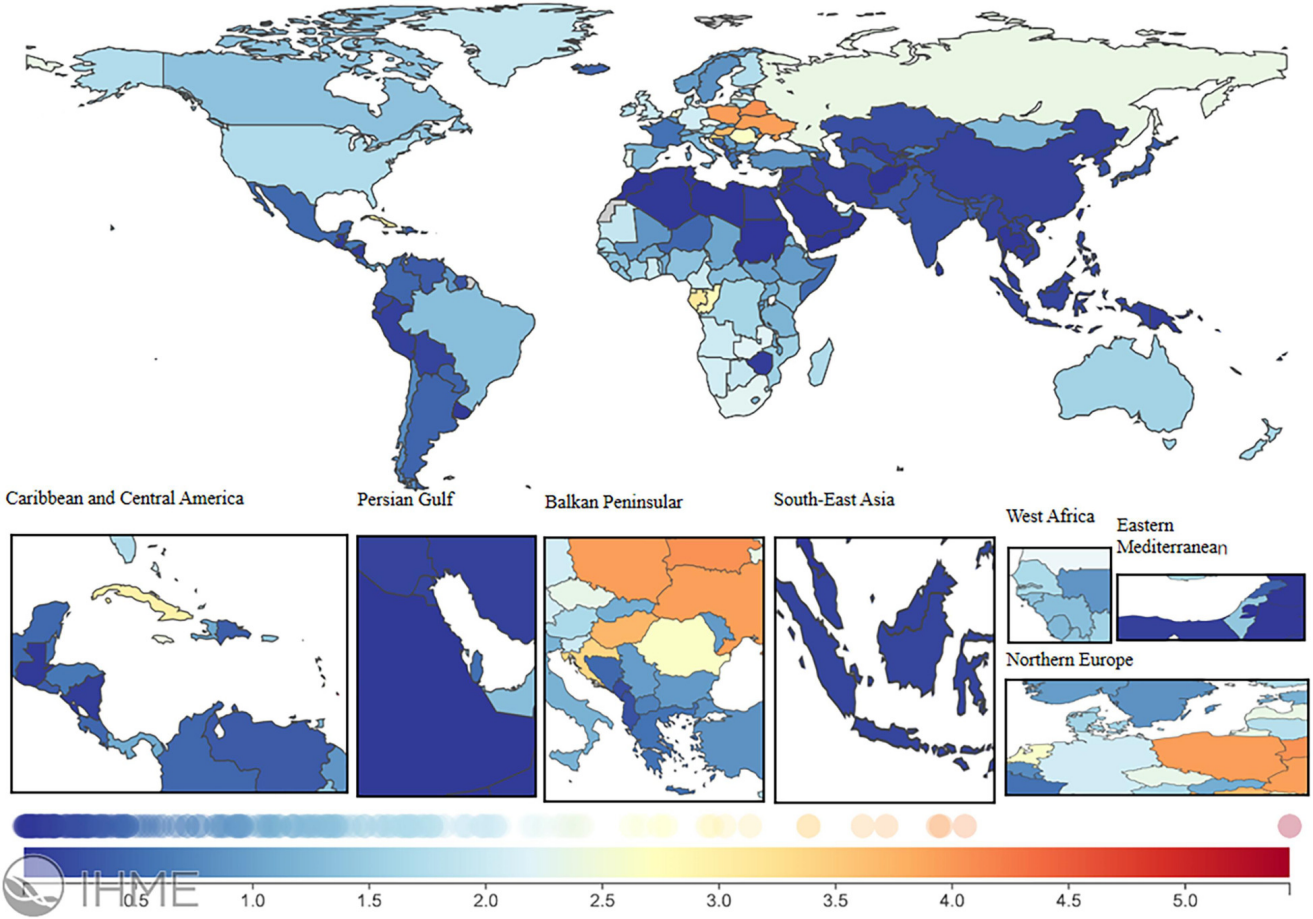
ASMR burden of LEPAD in 204 countries and territories in 2021. LEPAD, lower extremity peripheral artery disease; ASMR, age-standardized mortality rate; different colors represent different values, with blue to red indicating a progressively higher ASMR burden.

**Figure 4:**
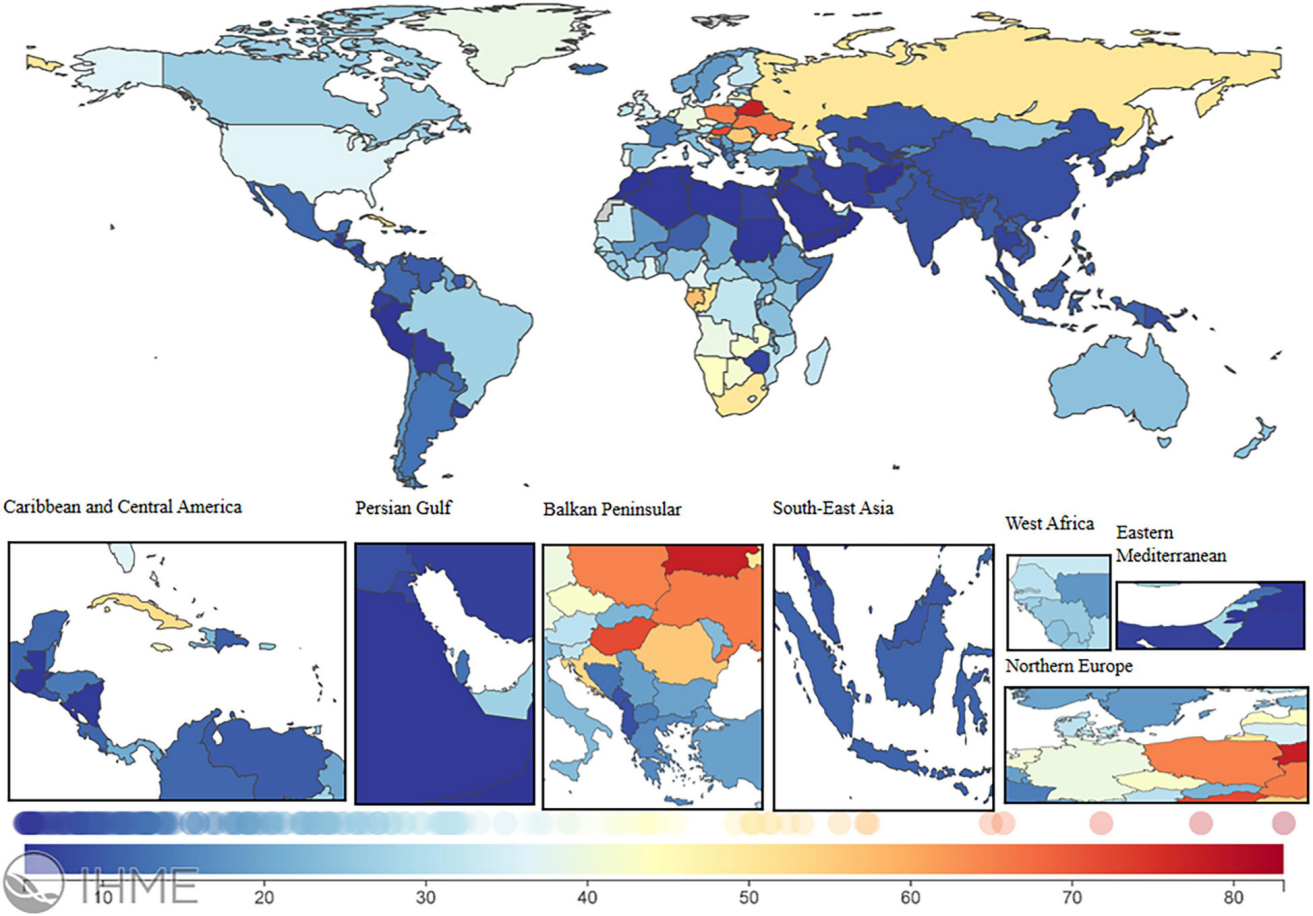
ASDR burden of LEPAD in 204 countries and regions in 2021. LEPAD, lower extremity peripheral arterial disease; ASDR, age-standardized disability-adjusted life years; different colors represent different values, with blue to red indicating a progressively higher ASDR burden.

In 2021, the global LEPAD ASIR was 115.44/100,000 [95% (UI) 100.04, 132.72] (Table 1). High SDI regions in North America and Western European countries had substantially higher ASIR than the global average (Figure 2). For instance, ASIRs in the United States and Denmark were 211.78 [95% (UI) 189.72, 235.81] and 192.8 [95% (UI) 165.92, 224.5], respectively (Supplementary Table S1). Significant differences in ASMRs for LEPAD were observed between countries, with the highest ASMRs occurring in Eastern and Central Europe (Figure 3). Belarus had the highest ASMR at 4.05 [95% (UI) 3.37, 4.81] (Supplementary Table S1), followed by Poland and Ukraine, both at 3.95 [95% (UI) 3.45, 4.46] (Supplementary Table S1). Furthermore, Eastern and Central Europe had the highest ASDRs globally (Figure 4), with an ASDR of 77.97 [95% (UI) 64.25, 93.17] in Belarus (Supplementary Table S1). In addition, sub-Saharan African countries reported ASDRs above the global average (Figure 4). ASIR, ASMR, and ASDR were lower in the Latin American Andes, Africa, and the Middle East (Figure 4).

At the national level, Afghanistan had the highest increase in ASIR between 1990 and 2021, with an average annual trend of 0.89%, followed by Egypt (APC 0.79%) and Saudi Arabia (APC 0.74%) (Supplementary Table S2). During the same period, Norway had the largest decrease in ASDR for LEPAD (APC −3.36%), followed by Sweden (APC −2.99%) (Supplementary Table S2). The largest increase in ASDR was observed in Georgia (APC 4.56%). Additionally, Georgia presented the most significant increase in ASMR (APC 12.94%) (Supplementary Table S2).

### Global and regional trends by age

**Figure 5:**
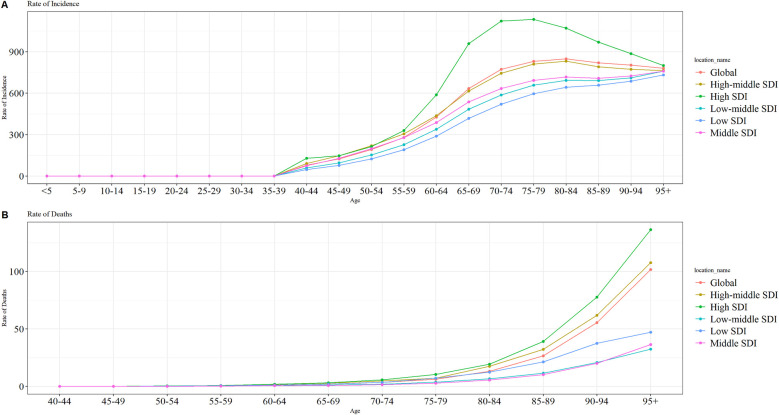
Trends in ASIRs and ASMRs by age group in 2021 globally and in different SDI regions. **(A)** global ASIR; **(B)** global ASMR. SDI, sociodemographic index; ASIR, age-standardized incidence rate; ASMR, age-standardized mortality rate.

In 2021, the global and regional LEPAD ASIR generally increased with age across all SDI regions (Figure 5A), with a low ASIR in individuals aged ˂ 40 years and a rapid increase in those aged ˃ 40 years, peaking in individuals aged 75–79 years (Figure 5A). In high SDI regions, ASIR in this age group reached 1,135.66 per 100,000 (Supplementary Table S3) and remained higher than that in other regions for individuals aged ˃ 60 years. In addition, ASIR and SDI in LEPAD were positively correlated across all age groups (Figure 5A).

In 2021, LEPAD ASMR increased with age both globally and in each SDI region (Figure 5B). ASMR was consistently higher in the low SDI regions than in the medium-low and medium SDI regions across different age groups. The ASMR of LEPAD both globally and in different SDI regions peaked in the 95+ age group, which was 136.32/100,000, 107.65/100,000, 36.39/100,000, 32.45/100,000, and 47.20/100,000 for low, medium-low, medium, medium-high, and high SDI, respectively (Supplementary Table S3).

### Analysis of temporal connectivity points

**Figure 6:**
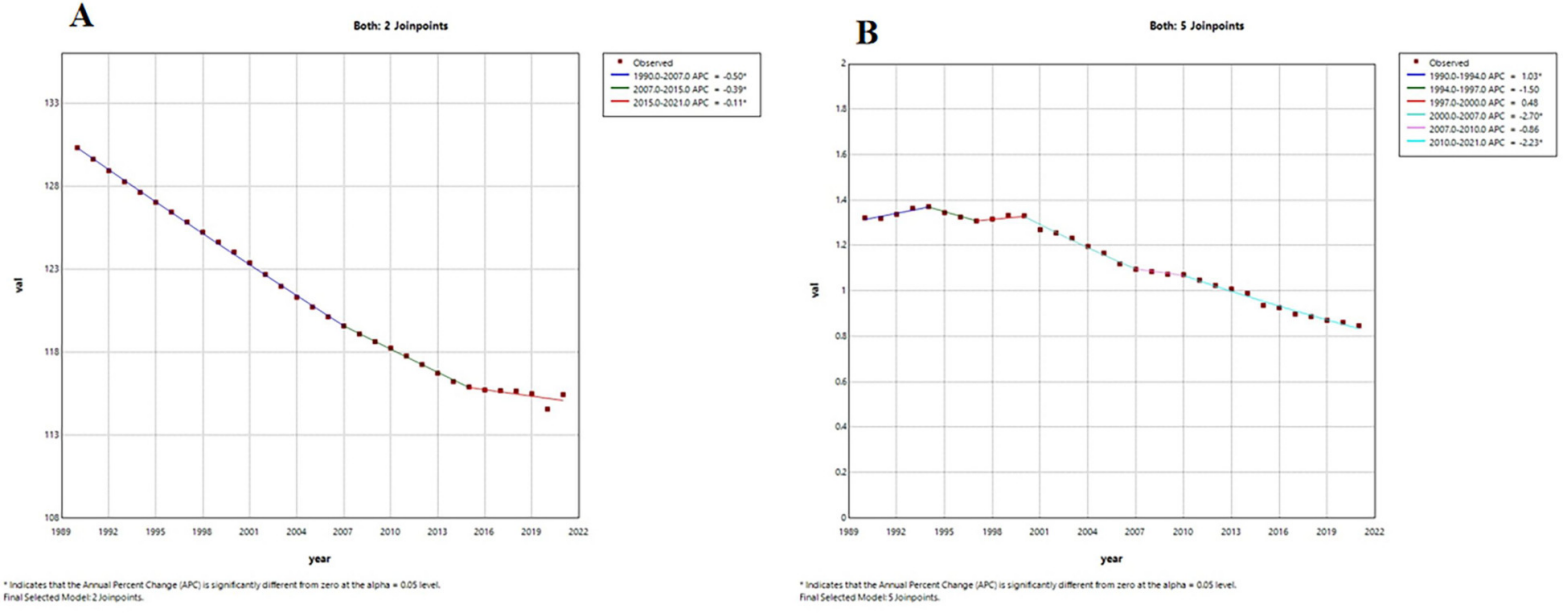
Trends in APC and AAPC (%) for global LEPAD ASIR, ASMR, 1990–2021. **(A)** global ASIR; **(B)** global ASMR. LEPAD, lower extremity peripheral arterial disease; ASIR, age-standardized incidence rate; ASMR, age-standardized mortality rate; APC, annual percentage change; AAPC, average annual percentage change.

From 1990 to 2021, the global ASIR for LEPAD showed a decreasing trend (Figure 6A), with AAPC of −0.40% (95% CI of −0.42, −0.38, *P* < 0.001) for ASIR and −1.46% (95% CI of, *P* < 0.001) for ASMR (Supplementary Table S4). The decline in ASIR was most pronounced from 1990 to 2007 (Figure 6A), with an APC of −0.50% (Supplementary Table S5). The ASMR of global LEPAD fluctuated from 1990 to 2021 but followed an overall downward trend (Figure 6B). The steepest decrease in ASMR occurred between 2000 and 2007, with an APC of −2.70% (Supplementary Table S5). However, between 1990 and 1994 and 1997–2000, ASMR showed an increasing trend, with APCs of 1.03% and 0.48%, respectively (Supplementary Table S5).

### Risk factors

**Figure 7:**
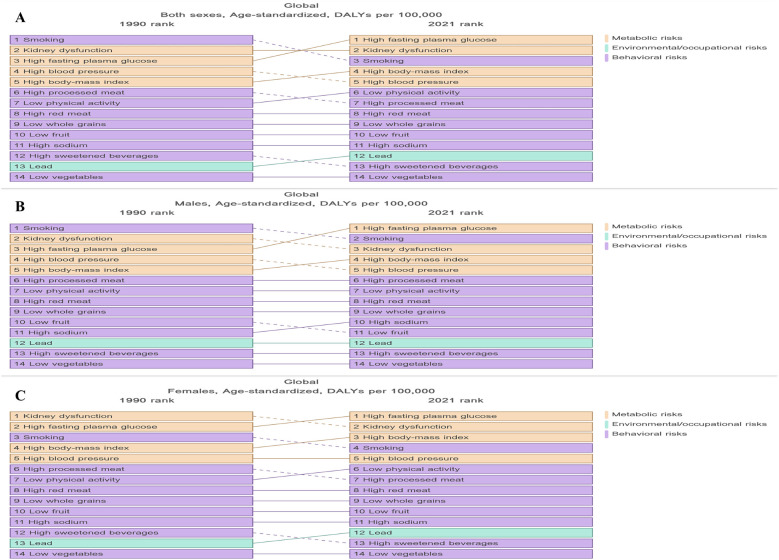
Risk factors associated with DALYs in global LEPAD populations stratified by sex, 1990–2021. **(A)** global total population ASDR; **(B)** global ASDR (men); **(C)** global ASDR (women). LEPAD, lower extremity peripheral artery disease; DALYs, disability-adjusted life years; ASDR, age-standardized disability-adjusted life years.

In 2021, metabolic factors remained the leading risk factors associated with LEPAD globally (Figure 7A). High fasting glucose level, renal dysfunction, smoking, high SDI, and hypertension were identified as major risk factors affecting LEPAD DALYs (Figure 7A). Complementary analysis using SEVs further revealed high low-density lipoprotein cholesterol (LDL-C) as the dominant metabolic risks, with a global age-standardized SEV of 45.3% in 2021, consistently ranking first among all metabolic exposures for LEPAD since 1990. In 1990, the first, second, and third major risk factors affecting LEPAD DALYs were smoking, renal dysfunction, and high fasting glucose levels, respectively (Figure 7A). However, by 2021, high fasting glucose levels and renal dysfunction surpassed smoking as the first and second risk factors, respectively (Figure 7A). Despite minimal decline in population exposure burden, high LDL-C maintained the highest SEV among metabolic risks throughout this period, indicating persistently widespread dyslipidemia exposure. In addition, the effects of risk factors on LEPAD DALYs varied according to sex. In 1990, smoking had the greatest impact on DALYs in men at 13.22/100,000 (Figure 7B), and renal dysfunction had the greatest impact on DALYs in female patients at 7.04/100,000 (Figure 7C). By 2021, high fasting glucose emerged as the leading risk factor globally for both sexes, with an ASDR of 7.68/100,000 and 5.92/100,000 in men and women, respectively (Figure 7A). SEV analysis identified high LDL-C as the leading metabolic risk exposure in both sexes. In addition, the LEPAD-related risk factors varied significantly by region. In low SDI regions, renal dysfunction was the primary mortality-related risk factor for LEPAD in men and women, with ASDRs of 3.82/100,000 and 3.09/100,000, respectively (Supplementary Figure S1). Conversely, high fasting glucose levels resulted in the highest LEPAD DALYs in high SDI regions, with an ASDR of 10.68/100,000 (Supplementary Figure S1). SEV analysis further demonstrated that high LDL-C was the predominant metabolic exposure in all SDI regions except low SDI areas, where high blood pressure ranked first (Supplementary Table S6).

### Disease incidence trend model prediction

**Figure 8:**
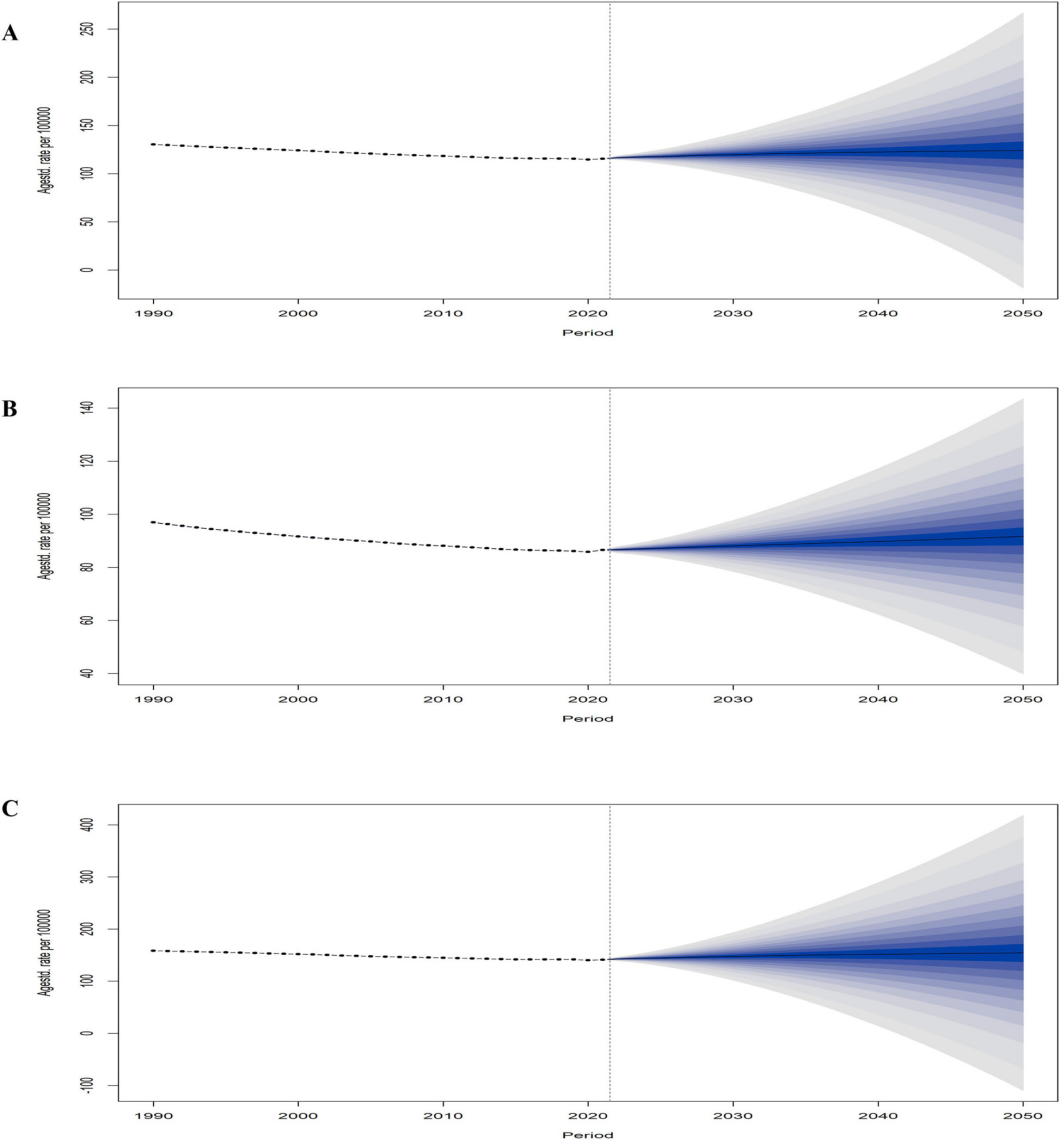
Global statistics and projections of LEPAD ASIR stratified by sex. **(A)** global ASIR in total population; **(B)** global ASIR (men); **(C)** global ASIR (women). LEPAD, lower extremity peripheral arterial disease; ASIR, age-standardized incidence rate.

The prediction results of the BAPC model indicated a gradual increase in the global LEPAD ASIR from 2022 to 2050 (Figure 8A), with consistently higher rates for women than for men (Figures 8B,C). In particular, the ASIR for men was 96.93 per 100,000 in 1990, declined over time, and reached the lowest value of 85.81 per 100,000 in 2020, and is projected to gradually increase to 91.66 per 100,000 in 2050 (Figure 8B). Similarly, the ASIR for women decreased from 158.44/100,000 in 1990 to 140.23/100,000 in 2020, which is expected to increase to 154.31/100,000 in 2050 (Figure 8C).

## Discussion

Our study is the first to report the status and trends of LEPAD burden at the global, regional, and national levels from 1990 to 2021 and predict the changes in the burden of disease over the next three decades. Additionally, we conducted a detailed analysis of the impacts of age, sex, risk factors, and the SDI on the ASR, which provides a reference basis for the development of national healthcare policies and the implementation of relevant diagnostic and treatment measures.

Between 1990 and 2021, total LEPAD cases and deaths increased worldwide; however, ASIR and ASMR decreased. Joinpoint analysis revealed a significant decline in ASIR in 1990–2000, whereas ASMR increased during this period, which could be attributed to the lack of awareness of LEPAD diagnosis among patients and healthcare professionals, leading to delayed diagnosis and treatment and thereby increasing the mortality rates. Despite technological progress in LEPAD diagnosis (12–14), LEPAD remains significantly underdiagnosed in high-risk populations—particularly among patients with established coronary heart disease (CHD). The large European survey EUROASPIRE (15) revealed that most of CHD patients without prior LEPAD diagnosis exhibited symptomatic LEPAD (e.g., intermittent claudication). This diagnostic gap impedes comprehensive cardiovascular risk management, as concurrent LEPAD portends higher mortality and mandates intensified secondary prevention. We thus emphasize systematic LEPAD screening for all CHD patients, irrespective of symptom presentation. The ASIR of LEPAD peaks at 75–79 years of age, whereas the ASMR continues to increase with age. The observed increase in total morbidity and mortality may be due to population growth and ageing rather than an actual increase in the proportion of individuals affected by LEPAD. The decrease in the age-standardized estimate of LEPAD may indicate ongoing improvements in medical prevention and management strategies.

LEPAD, an atherosclerotic vascular disease, is highly prevalent in high-income countries. However, the burden of LEPAD has been rapidly increasing in recent years in many low-income and middle-income countries, which may be influenced by changes in lifestyle and the environment due to urbanization and economic development (16–18). Our findings highlighted that age-standardized morbidity, age-standardized mortality, and age-standardized DALYs were highest in high SDI regions in 2021, aligning with the findings of previous studies (19). The SDI is strongly associated with factors such as socioeconomic status, education, and dietary habits, which significantly impact the burden of cardiovascular disease (20, 21). Our findings demonstrated that the ASIR and ASMR of the LEPAD were highest in high SDI regions, and the most significant increase in these rates was observed in low SDI regions. A plausible explanation is that the predisposing factors and diagnosis rates increase with economic development in high SDI regions. Rapid urbanization and industrialization have exacerbated lifestyle changes, including unhealthy diets and a lack of physical activity, which contribute to the occurrence of LEPAD. Additionally, factors such as low income, lack of access to medical care, and low levels of education may potentially may contribute to the increased disease burden of LEPAD in low SDI regions (22, 23). The low prevalence of LEPAD in low SDI areas may be partly due to the lack of medical resources, resulting in delayed or inadequate treatment for mild or asymptomatic cases (24). Therefore, prioritizing early diagnosis and appropriate management is crucial in areas with scarce medical resources.

LEPAD morbidity and mortality are higher in the older age groups, with the highest global prevalence observed in those aged 75–79 years at 831.29/100,000 (95% UI 562.11, 1162.93) in 2021. Notably, prevalence rates varied by sex (1015.77/100,000 [686.85, 1424.99] for women and 608.83/100,000 [415.97, 850.09] for men). This age-related trend can be attributed to the accompanying geriatric syndromes, such as weakness, muscle loss, malnutrition, and functional decline in older adults, which may mask symptoms associated with LEPAD, often delaying diagnosis until the disease has progressed to an advanced stage. The common age group for LEPAD onset observed in the present study aligns with the latest guidelines of the Global Study of Lower Extremity Peripheral Arterial Disease (1). Our findings indicated that the prevalence of LEPAD was higher in women than in men, which may be related to factors such as lower pain threshold in women (25, 26), greater tendency to seek medical help (27), and sex hormone differences (28–30). Menopause is closely associated with an increased risk of LEPAD (31), suggesting that differences in hormone levels before and after menopause play an important role in LEPAD development. However, despite the higher prevalence in women, the DALYs for LEPAD were similar in both sexes, and the total DALYs attributable to modifiable risk factors were higher in men than in women, suggesting that men experience a more severe burden of disability, which may be attributed to the higher prevalence of major risk factors (e.g., smoking, hypertension, diabetes mellitus) (32) and delayed diagnosis. These findings underscore the need for sex-specific approaches in LEPAD management.

Risk factor analyses revealed that high fasting glucose, renal dysfunction, smoking, high BMI, and hypertension were key contributors to LEPAD-related deaths and disabilities, with high fasting glucose accounting for 36% of the global DALYs in 2021. Critically, our complementary SEV assessment identified persistently high LDL-C exposure (45.3% globally in 2021) as the dominant metabolic risk burden. This result is consistent with the pathological chain of dyslipidemia-atherosclerosis-LEPAD clearly confirmed by clinical studies (33, 34), but it is not fully reflected in the attribution of DALYs. It may be due to the conservative attribution effect (RR value) of high LDL-C on LEPAD in the GBD model, or its health loss is combined to the endpoint of cardiovascular disease. It is recommended that the future LEPAD prevention and control guidelines include blood lipid management into the core strategy. The impact of these risk factors varies according to the SDI level (Figure 7), highlighting the need for targeted prevention strategies based on SDI zones. For example, In high SDI regions, integrated management of glucose and lipid parameters should be prioritized due to substantial LDL-C exposure burden (SEV >50%), with structured programs such as diabetes-lipid integrated care recommended for implementation (35); For low SDI regions, high blood pressure management constitutes the primary focus (dominant SEV: 35.78%) due to healthcare resource limitations, with lipid control implemented as secondary prevention where feasible (17). In conclusion, LEPAD prevention must address triad metabolic risks: diabetes mellitus, renal dysfunction, and high LDL-C. Individuals exposed to these risks require education campaigns emphasizing lipid profile monitoring alongside glucose/blood pressure control, particularly in regions with elevated SEV burden.

Furthermore, it is crucial to recognize that LEPAD is not an isolated vascular condition but a key manifestation of systemic atherosclerosis, often coexisting with coronary artery disease and cerebrovascular disease (36). Patients with polyvascular atherosclerosis face significantly higher risks of major adverse cardiovascular events and mortality. The shared pathophysiological underpinnings—such as endothelial dysfunction, lipid accumulation, and inflammatory pathways—imply that preventive strategies for LEPAD are largely congruent with those for coronary artery disease and cerebrovascular disease (37). These include aggressive management of modifiable risk factors like dyslipidemia (particularly LDL-C control), hypertension, diabetes, smoking cessation, and antiplatelet therapy (38). Therefore, integrated cardiovascular risk reduction programs that address all atherosclerotic territories simultaneously are warranted, especially in high-risk populations. Early detection of LEPAD should prompt comprehensive vascular assessment and intensified preventive measures to mitigate overall atherosclerotic burden.

The BAPC model predicted an increase in the total number of LEPAD cases over the next 30 years (Supplementary Table S7), likely because of the global population growth and aging and an increase in the prevalence of LEPAD (Figure 8) (39). The results of the model predictions indicate a general upward trend in the ASIR for approximately three decades after 2021. However, the ASIR for LEPAD shows a downward trend from 1990 to 2021. Apparently, this decline could be attributed to disease prevention, screening, and anti-platelet therapy. Nevertheless, the concurrent increase in the number of patients with diabetes, renal dysfunction, and cigarette smoking in recent years has largely offset these benefits, potentially exacerbating the future burden of LEPAD (5).

This study had certain limitations. Firstly, the GBD primarily compiles data from different national and regional studies and publications rather than direct national studies, which may impact completeness and timeliness, especially in areas with low SDI. Although the GBD study used rigorous statistical methods to address these uncertainties, the results should be interpreted as the best available estimates based on the current evidence. Furthermore, variations in disease management, including diagnosis, recording, and reporting across countries and regions, may have affected data comparability. Such differences may lead to under-reporting, although the true burden is often only available through detailed calculations in countries or regions with a higher disease prevalence.

## Conclusion

The total burden of LEPAD has increased globally from 1990 to 2021; however, age-standardized rates have declined, which is a key factor influencing the prevalence of LEPAD and is influenced by global population growth, aging, and sex. The ASIR and ASMR for the LEPAD showed an overall upward trend with increasing age. Although ASIR for LEPAD was lower in men than in women, the ASMR and ASDR were substantially higher. High fasting glucose and renal dysfunction drive LEPAD DALYs, yet SEV metrics expose high LDL-C as the dominant metabolic exposure burden, signalling unmet prevention priorities. The burden of LEPAD is projected to increase through 2050, highlighting the need for targeted health education, regular screening, and multidisciplinary healthcare to reduce the global burden of LEPAD.

## Data Availability

The datasets presented in this study can be found in online repositories. The names of the repository/repositories and accession number(s) can be found in the article/Supplementary Material.
