# Supplementary material for: Global, regional, and national burdens of lower extremity peripheral arterial disease from 1990 to 2021 and projections to 2050: global burden of disease study 2021
Source: Front Cardiovasc Med. 2025 Oct 7;12:1592322. doi: 10.3389/fcvm.2025.1592322 (PMC12537751; doi:10.3389/fcvm.2025.1592322)
Supplement: Supplementary file 1 [file Datasheet1.pdf]

Supplementary Table 1 ASIR, ASMR, and ASDR of LEPAD in different countries in 2021

Country	ASIR(95%UI)	ASMR(95%UI)	ASDR(95%UI)
Afghanistan	91.72(79.00,106.55)	0.02(0.01,0.03)	5.13(2.59,9.76)
Angola	82.96(71.36,96.90)	2.01(1.17,3.50)	38.86(24.31,64.37)
Albania	97.26(83.56,113.02)	0.27(0.16,0.41)	8.54(5.36,12.88)
Andorra	151.56(131.06,175.94)	1.18(0.68,1.77)	23.38(15.21,33.25)
United Arab Emirates	93.31(80.40,107.75)	1.31(0.93,1.77)	27.15(20.45,34.61)
Argentina	134.52(116.53,156.43)	0.42(0.37,0.47)	12.81(9.41,18.71)
Armenia	99.20(84.52,115.36)	2.40(1.99,2.82)	43.25(35.47,51.95)
American Samoa	140.03(121.31,163.36)	0.76(0.51,1.34)	22.97(16.61,33.00)
Antigua and Barbuda	85.37(72.88,100.08)	1.76(1.57,1.94)	31.37(27.82,35.92)
Australia	105.20(90.18,121.41)	1.58(1.31,1.77)	24.11(20.56,28.54)
Austria	160.50(138.56,185.44)	1.67(1.41,1.83)	30.96(26.22,38.14)
Azerbaijan	93.09(80.50,108.28)	0.37(0.17,0.71)	11.42(6.28,19.35)
Burundi	71.66(61.96,82.82)	0.93(0.36,2.14)	18.98(8.80,37.79)
Belgium	164.04(141.77,190.60)	0.79(0.65,0.88)	18.92(14.50,26.43)
Benin	77.24(66.38,89.50)	1.10(0.46,2.32)	21.32(10.53,41.42)
Burkina Faso	72.27(62.51,84.45)	1.07(0.31,2.94)	20.51(7.83,51.50)
Bangladesh	77.49(66.82,90.61)	0.35(0.20,0.59)	8.86(5.52,13.86)
Bulgaria	98.11(84.39,113.96)	0.92(0.75,1.11)	19.23(15.16,24.93)
Bahrain	108.39(93.60,126.52)	1.35(1.01,1.84)	26.35(19.43,35.85)
Bahamas	82.29(70.12,95.99)	2.03(1.68,2.41)	37.14(30.53,44.29)
Bosnia and Herzegovina	115.01(99.38,132.75)	0.46(0.32,0.65)	12.74(8.99,18.42)
Belarus	96.79(83.45,112.23)	4.05(3.37,4.81)	77.97(64.25,93.17)
Belize	82.95(71.41,96.44)	0.50(0.43,0.57)	12.30(9.96,16.61)
Bermuda	77.45(66.27,90.09)	3.02(2.49,3.68)	45.43(38.73,55.09)
Bolivia (Plurinational State of)	73.14(62.91,85.45)	0.15(0.10,0.21)	6.12(4.04,9.64)
Brazil	88.48(76.16,102.76)	1.32(1.15,1.44)	27.03(23.72,31.39)
Barbados	85.55(73.33,100.03)	5.45(4.49,6.54)	83.10(68.10,100.44)
Brunei Darussalam	116.60(100.05,136.06)	0.58(0.45,0.73)	13.89(10.30,19.27)
Bhutan	73.85(64.14,85.63)	0.26(0.12,0.40)	7.57(4.30,11.90)
Botswana	96.69(83.37,112.47)	1.92(1.22,2.70)	41.65(28.58,57.49)
Central African Republic	82.35(70.80,97.08)	1.28(0.59,2.41)	27.77(14.06,47.45)
Canada	169.05(146.43,194.20)	1.37(1.17,1.51)	26.36(22.38,32.63)
Switzerland	151.55(130.66,175.81)	0.98(0.80,1.11)	20.27(15.92,26.91)
Chile	134.72(116.43,155.94)	0.77(0.66,0.84)	17.06(13.73,23.08)
Cte d'Ivoire	79.21(68.01,91.79)	1.55(0.69,3.21)	29.53(13.83,57.36)
Cameroon	78.00(67.00,90.27)	1.98(1.03,3.51)	37.03(20.48,63.51)
Democratic Republic of the Congo	78.59(67.85,92.25)	1.61(0.73,3.16)	31.81(16.13,58.64)
Congo	81.73(70.46,95.10)	2.77(1.82,4.05)	51.18(36.34,73.00)
Cook Islands	124.28(106.74,144.35)	0.25(0.17,0.42)	12.50(8.11,19.38)

Colombia	92.12(78.80,107.67)	0.44(0.37,0.53)	11.26(8.67,16.00)
Comoros	73.57(63.43,85.23)	0.95(0.25,2.09)	18.58(6.96,35.86)
Cabo Verde	79.27(68.70,92.70)	2.19(1.17,3.66)	37.48(21.51,58.47)
Costa Rica	96.38(82.20,112.40)	0.45(0.37,0.51)	11.18(8.55,15.64)
Cuba	96.81(82.43,112.21)	2.97(2.56,3.37)	52.42(45.20,60.22)
Cyprus	171.37(147.56,197.97)	1.66(1.25,2.23)	27.79(21.08,36.59)
Czechia	103.69(89.44,119.90)	2.34(1.94,2.73)	42.57(35.49,51.37)
Germany	166.21(142.84,192.35)	2.04(1.72,2.26)	39.54(33.78,46.86)
Djibouti	77.54(65.96,90.30)	1.73(0.89,3.05)	32.46(18.51,53.24)
Dominica	86.54(73.75,100.03)	1.63(1.27,2.03)	29.77(23.84,37.05)
Denmark	192.80(165.92,224.50)	1.58(1.33,1.77)	31.19(25.64,40.37)
Dominican Republic	82.10(70.55,96.09)	0.37(0.26,0.51)	10.31(7.36,14.79)
Algeria	98.21(84.67,114.95)	0.05(0.03,0.09)	5.65(3.06,10.15)
Ecuador	75.24(64.40,87.37)	0.20(0.16,0.25)	6.64(4.37,10.31)
Eritrea	71.77(61.64,83.11)	1.19(0.61,2.44)	23.15(12.95,43.41)
Spain	146.40(125.32,170.27)	1.24(1.05,1.40)	24.00(19.60,30.00)
Estonia	96.32(82.38,111.90)	1.17(0.99,1.37)	22.63(18.95,27.73)
Ethiopia	65.93(56.53,76.75)	0.94(0.46,1.61)	18.29(10.22,29.71)
Finland	156.09(135.32,181.23)	1.74(1.45,1.97)	31.89(26.77,39.37)
Fiji	136.16(117.52,158.77)	0.30(0.20,0.44)	13.69(8.87,22.51)
France	145.95(126.39,169.19)	0.54(0.45,0.61)	14.17(10.45,20.59)
Micronesia (Federated States of)	128.40(111.03,150.07)	0.31(0.20,0.44)	15.41(10.18,23.81)
Gabon	78.88(68.26,90.73)	3.13(2.21,4.48)	57.21(41.09,79.98)
United Kingdom	162.46(140.61,187.32)	1.98(1.71,2.14)	34.85(29.90,42.67)
Georgia	101.45(86.85,117.10)	0.86(0.72,1.00)	21.12(16.83,26.96)
Ghana	76.86(66.30,89.69)	2.01(1.27,3.12)	36.30(24.95,54.14)
Guinea	76.76(66.22,89.22)	1.32(0.53,2.90)	25.92(11.93,52.32)
Gambia	79.16(67.97,91.67)	1.67(0.81,3.43)	31.42(16.99,59.73)
Guinea-Bissau	75.41(65.00,87.68)	1.36(0.71,2.72)	26.92(14.90,49.86)
Equatorial Guinea	80.60(69.46,93.35)	2.74(1.55,4.34)	50.34(29.69,77.89)
Greece	173.98(150.64,201.27)	0.55(0.47,0.61)	15.87(11.64,24.04)
Grenada	88.60(76.11,103.03)	2.95(2.56,3.31)	49.18(42.83,56.09)
Greenland	183.33(158.66,213.12)	1.90(1.45,2.47)	38.76(29.87,50.36)
Guatemala	91.67(78.72,107.34)	0.06(0.05,0.07)	5.63(3.14,10.27)
Guam	110.68(96.12,127.67)	0.07(0.06,0.09)	9.18(5.48,15.64)
Guyana	89.27(76.64,103.72)	0.87(0.70,1.08)	19.90(15.99,25.59)
Honduras	93.34(80.34,107.49)	0.60(0.43,0.80)	13.74(10.30,18.73)
Croatia	101.90(87.85,118.63)	3.39(2.89,3.90)	53.29(45.55,62.66)
Haiti	85.28(73.43,99.94)	1.05(0.61,1.62)	22.35(14.06,33.72)
Hungary	99.35(85.28,115.50)	3.72(3.12,4.33)	71.80(60.18,86.63)
Indonesia	128.57(111.14,149.26)	0.15(0.09,0.22)	10.90(6.35,18.70)
India	84.97(73.21,98.57)	0.25(0.16,0.41)	8.45(5.46,13.08)
Ireland	156.09(134.28,182.04)	1.79(1.47,2.05)	31.59(26.27,39.54)

Iran (Islamic Republic of)	92.14(79.43,106.40)	0.12(0.10,0.15)	6.42(3.93,10.53)
Iraq	113.33(97.80,132.25)	0.14(0.10,0.19)	8.22(5.17,13.53)
Iceland	153.04(131.26,176.63)	0.42(0.34,0.48)	12.55(8.66,19.18)
Israel	160.03(137.48,187.32)	1.41(1.18,1.59)	28.02(22.77,35.18)
Italy	161.78(140.17,186.33)	1.17(0.97,1.33)	23.07(18.36,30.76)
Jamaica	90.51(76.85,105.57)	2.41(1.91,3.03)	43.94(34.79,55.40)
Jordan	106.16(91.60,123.37)	0.05(0.04,0.06)	5.79(3.13,10.69)
Japan	113.02(97.48,130.17)	0.35(0.28,0.40)	9.66(7.00,14.13)
Kazakhstan	99.79(85.08,116.05)	0.24(0.19,0.29)	9.04(6.34,13.82)
Kenya	76.32(65.66,88.35)	1.33(0.82,2.09)	24.96(16.71,36.90)
Kyrgyzstan	87.25(75.04,101.90)	0.62(0.51,0.74)	15.51(12.16,19.96)
Cambodia	127.05(109.86,147.85)	0.09(0.04,0.16)	9.33(4.97,16.92)
Kiribati	150.97(129.69,176.79)	0.28(0.12,0.53)	16.88(9.24,27.39)
Saint Kitts and Nevis	84.51(72.89,98.24)	3.62(3.08,4.18)	57.38(48.92,66.77)
Republic of Korea	100.88(86.62,116.11)	0.29(0.23,0.35)	7.94(5.55,11.89)
Kuwait	98.61(85.05,115.19)	0.16(0.12,0.19)	7.30(4.79,11.66)
Lao People's Democratic Republic	122.68(106.04,143.06)	0.15(0.10,0.22)	10.73(6.18,18.55)
Lebanon	118.68(101.87,137.39)	0.40(0.31,0.50)	12.06(8.56,17.55)
Liberia	78.90(67.59,92.34)	1.39(0.49,3.01)	25.88(10.95,52.19)
Libya	104.03(89.34,121.33)	0.02(0.01,0.05)	5.58(2.74,10.45)
Saint Lucia	92.11(78.76,107.11)	3.38(2.78,4.02)	51.35(43.08,61.40)
Sri Lanka	124.02(106.51,144.68)	0.09(0.06,0.13)	8.59(4.89,15.50)
Lesotho	94.13(80.46,109.39)	1.26(0.52,2.01)	31.56(15.14,50.57)
Lithuania	90.68(78.16,105.23)	1.77(1.52,2.02)	34.92(29.52,41.17)
Luxembourg	169.35(146.48,195.29)	0.72(0.62,0.81)	18.17(13.65,25.92)
Latvia	95.43(82.08,110.00)	2.36(1.98,2.75)	43.66(37.04,51.89)
Morocco	93.35(80.19,108.59)	0.04(0.02,0.05)	5.29(2.75,9.77)
Monaco	151.50(131.20,175.55)	1.64(1.18,2.23)	30.65(22.68,41.86)
Republic of Moldova	99.12(84.60,115.69)	0.93(0.79,1.07)	23.08(19.36,28.04)
Madagascar	71.94(62.26,83.73)	1.70(0.96,3.53)	31.93(19.58,62.57)
Maldives	113.72(98.84,133.07)	0.08(0.05,0.11)	7.59(4.05,14.01)
Mexico	90.76(77.78,105.02)	0.46(0.40,0.51)	11.52(8.83,16.12)
Marshall Islands	127.46(109.84,150.29)	0.23(0.13,0.35)	12.89(7.84,21.08)
North Macedonia	110.47(94.64,128.20)	0.68(0.43,1.03)	14.41(9.56,20.91)
Mali	73.25(63.34,84.68)	0.87(0.32,1.89)	17.79(7.98,33.49)
Malta	163.78(140.78,187.74)	1.48(1.23,1.71)	28.55(23.55,36.29)
Myanmar	122.76(105.76,143.81)	0.14(0.10,0.20)	10.27(6.24,17.73)
Montenegro	118.72(101.69,137.47)	0.24(0.18,0.32)	8.97(5.99,14.00)
Mongolia	86.14(74.44,100.51)	1.23(0.78,1.87)	24.16(16.40,33.54)
Northern Mariana Islands	114.36(99.73,133.17)	0.30(0.21,0.40)	12.81(8.88,19.22)
Mozambique	79.15(67.78,91.97)	1.54(0.74,3.33)	30.86(15.73,61.88)
Mauritania	72.81(62.29,84.63)	1.92(1.11,3.51)	33.29(20.43,60.03)
Mauritius	130.91(113.46,153.25)	1.02(0.91,1.12)	25.67(21.23,33.32)

Malawi	82.79(71.69,96.02)	1.07(0.39,2.25)	22.45(10.31,43.45)
Malaysia	121.76(105.03,142.00)	0.14(0.11,0.17)	9.57(5.68,16.36)
Namibia	85.60(73.97,99.60)	2.06(1.39,2.88)	43.59(30.02,58.75)
Niger	70.09(60.76,80.76)	0.44(0.07,1.33)	10.43(3.75,24.71)
Nigeria	75.33(64.74,87.67)	1.35(0.63,2.85)	23.82(12.52,46.34)
Nicaragua	91.91(78.20,106.96)	0.09(0.07,0.12)	5.93(3.45,10.11)
Niue	137.80(118.28,161.04)	0.25(0.19,0.35)	13.95(9.23,22.05)
Netherlands	144.32(124.85,166.83)	2.62(2.22,2.92)	41.04(35.33,48.44)
Norway	153.48(132.99,176.59)	0.96(0.79,1.06)	19.53(15.24,26.42)
Nepal	84.75(72.63,98.86)	0.29(0.14,0.49)	8.61(4.96,13.42)
Nauru	129.60(111.01,151.70)	0.39(0.22,0.67)	16.68(10.68,25.37)
New Zealand	107.44(92.60,124.70)	1.65(1.34,1.87)	24.86(20.74,29.51)
Oman	86.06(74.10,99.85)	0.06(0.03,0.09)	5.25(2.76,9.12)
Pakistan	89.13(76.80,104.11)	0.32(0.17,0.56)	9.86(5.91,15.82)
Panama	87.57(74.76,101.16)	1.08(0.83,1.29)	20.40(16.33,25.65)
Peru	69.74(60.00,81.14)	0.12(0.09,0.17)	5.21(3.34,8.57)
Philippines	123.84(106.98,144.23)	0.21(0.17,0.25)	11.82(7.55,19.42)
Palau	120.67(103.39,140.44)	0.27(0.19,0.38)	12.55(8.84,19.63)
Papua New Guinea	103.42(89.11,120.19)	0.09(0.04,0.17)	8.45(4.40,14.79)
Poland	100.77(86.83,116.47)	3.95(3.45,4.46)	64.96(56.45,73.59)
Puerto Rico	90.97(78.26,105.32)	1.44(1.18,1.71)	27.03(22.47,32.70)
Democratic People's Republic of Korea	119.47(103.69,138.84)	0.10(0.06,0.15)	8.76(4.86,15.97)
Portugal	153.12(131.95,178.32)	2.31(1.93,2.57)	38.79(32.94,46.19)
Paraguay	90.55(77.00,105.54)	0.43(0.32,0.55)	11.59(8.67,16.07)
Palestine	105.88(91.10,123.16)	0.03(0.02,0.04)	5.54(2.87,10.56)
Qatar	92.71(79.89,107.36)	0.52(0.33,0.84)	12.83(8.27,19.53)
Romania	93.19(80.51,108.05)	2.67(2.27,3.07)	55.59(46.99,65.30)
Russian Federation	113.39(97.74,131.16)	2.37(2.16,2.57)	50.17(44.94,56.72)
Rwanda	82.26(70.91,95.79)	0.89(0.16,2.10)	18.18(5.68,38.14)
Saudi Arabia	92.95(80.24,108.71)	0.05(0.04,0.07)	5.56(3.05,9.79)
Senegal	82.45(70.55,95.16)	1.68(0.73,3.56)	30.96(15.20,60.40)
Singapore	93.48(80.14,107.81)	0.72(0.59,0.81)	13.47(10.99,17.25)
Solomon Islands	121.10(105.57,140.40)	0.17(0.06,0.31)	11.10(6.03,18.91)
Sierra Leone	78.64(67.40,91.25)	1.24(0.51,2.81)	24.62(11.34,51.91)
El Salvador	89.58(76.65,104.38)	0.36(0.24,0.53)	9.64(6.69,14.20)
San Marino	154.44(133.19,178.65)	0.66(0.39,1.03)	15.92(10.35,24.06)
Somalia	77.44(66.82,90.15)	0.46(0.11,1.33)	12.35(4.81,28.09)
Serbia	107.67(92.18,125.64)	0.95(0.76,1.18)	18.96(14.81,24.08)
South Sudan	73.28(62.91,85.58)	0.95(0.33,2.19)	19.67(8.41,39.73)
Sao Tome and Principe	79.13(68.10,91.66)	1.77(0.97,3.43)	31.21(18.50,56.28)
Suriname	94.70(81.31,110.36)	0.29(0.20,0.41)	10.08(7.01,14.91)
Slovakia	91.87(79.44,106.46)	1.12(0.90,1.43)	22.12(17.31,28.24)
Slovenia	94.62(81.75,109.81)	1.99(1.56,2.46)	31.81(25.56,39.74)

Sweden	158.66(137.17,183.02)	0.84(0.68,0.96)	17.93(13.67,25.18)
Eswatini	88.36(76.30,102.02)	1.82(1.10,2.91)	44.35(27.44,71.52)
Seychelles	132.80(114.59,154.14)	0.19(0.09,0.28)	11.67(6.83,19.63)
Syrian Arab Republic	100.28(86.50,117.66)	0.04(0.03,0.06)	5.38(2.82,9.89)
Chad	73.39(63.12,85.03)	1.04(0.35,2.32)	20.72(8.48,41.54)
Togo	81.47(70.13,94.47)	1.49(0.75,2.98)	28.74(15.84,54.16)
Thailand	108.00(92.17,125.66)	0.04(0.03,0.06)	6.83(3.59,12.44)
Tajikistan	84.29(72.84,98.28)	0.20(0.12,0.33)	7.59(4.78,12.09)
Tokelau	122.63(106.15,143.22)	0.25(0.16,0.42)	12.22(7.99,18.90)
Turkmenistan	88.83(76.54,103.40)	0.34(0.25,0.47)	11.17(7.86,16.03)
Timor-Leste	116.32(100.08,135.65)	0.11(0.07,0.17)	9.25(5.22,16.91)
Tonga	133.96(114.74,158.40)	0.22(0.13,0.35)	12.70(7.94,20.20)
Trinidad and Tobago	93.29(79.42,108.49)	1.27(0.98,1.60)	25.57(20.46,32.84)
Tunisia	102.99(88.48,119.68)	0.05(0.03,0.10)	5.78(3.10,10.38)
Trkiye	97.78(83.56,113.70)	0.85(0.64,1.07)	19.11(14.70,24.75)
Tuvalu	127.72(109.90,148.78)	0.23(0.15,0.35)	13.13(8.19,21.60)
Taiwan (Province of China)	127.46(113.93,144.25)	0.04(0.03,0.04)	7.87(4.09,14.62)
United Republic of Tanzania	79.77(68.07,93.29)	1.21(0.42,2.73)	23.85(10.43,48.11)
Uganda	76.59(65.76,89.34)	1.11(0.55,2.24)	22.30(12.33,41.17)
Ukraine	106.35(91.87,123.46)	3.94(3.05,4.94)	65.74(51.64,82.88)
Uruguay	130.95(112.77,150.68)	0.06(0.05,0.07)	6.98(3.80,12.90)
United States of America	211.78(189.72,235.81)	1.72(1.48,1.87)	36.72(31.54,44.77)
Uzbekistan	84.82(73.17,99.74)	0.11(0.09,0.14)	6.26(3.94,10.48)
Saint Vincent and the Grenadines	85.89(73.75,100.76)	1.30(1.15,1.48)	23.66(20.46,28.37)
Venezuela (Bolivarian Republic of)	94.83(80.94,109.36)	0.34(0.25,0.42)	9.95(7.28,14.65)
United States Virgin Islands	85.56(73.07,99.47)	2.04(1.35,2.94)	32.15(23.01,43.36)
Viet Nam	123.59(106.60,143.81)	0.14(0.09,0.21)	9.74(5.82,16.37)
Vanuatu	122.34(105.89,143.12)	0.15(0.06,0.25)	10.92(5.74,18.80)
Samoa	135.24(116.34,158.85)	0.21(0.12,0.38)	12.85(7.61,21.76)
Yemen	94.00(81.08,109.73)	0.03(0.01,0.05)	5.35(2.77,9.92)
South Africa	99.28(85.75,114.94)	2.25(1.98,2.53)	50.13(43.24,57.71)
Zambia	73.98(63.67,86.41)	2.21(1.02,4.35)	42.06(21.15,83.26)
Zimbabwe	98.37(84.61,114.31)	0.09(0.06,0.12)	7.28(4.49,12.50)
Egypt	95.38(82.20,111.24)	0.12(0.09,0.15)	6.75(4.16,11.37)
Sudan	99.12(85.33,115.53)	0.03(0.02,0.04)	5.59(2.87,10.54)
China	112.66(97.75,130.73)	0.13(0.10,0.16)	8.36(4.87,14.33)

LEPAD, lower extremity peripheral arterial disease; ASIR, age-standardised incidence rate; ASMR, age-standardised mortality rate; ASDR, age-standardised disability-adjusted life years; UI, uncertainty interval.

Supplementary Table 2 **APC of LEPAD ASIR, ASMR, ASDR in different countries**

Country	APC		
	ASIR	ASMR	ASDR
Afghanistan	0.008893	0.08127	0.007974
Angola	0.001757	0.017424	0.013196
Albania	0.001437	0.011619	0.002451
Andorra	-0.00635	-0.01344	-0.01315
United Arab Emirates	0.004348	0.008972	0.004114
Argentina	-0.00409	-0.0034	-0.00569
Armenia	0.002664	0.000416	-0.00149
American Samoa	0.005598	-0.00436	-0.00313
Antigua and Barbuda	0.002809	0.004144	0.002628
Australia	-0.01027	-0.02552	-0.02534
Austria	-0.00399	-0.02057	-0.01846
Azerbaijan	0.00371	0.015243	0.00852
Burundi	-0.00082	-0.00074	-0.00321
Belgium	-0.0055	-0.00825	-0.00873
Benin	0.001675	0.014907	0.010375
Burkina Faso	0.00114	0.018026	0.013203
Bangladesh	0.00153	0.023425	0.008078
Bulgaria	0.001093	0.01368	0.01076
Bahrain	0.004876	0.037479	0.022942
Bahamas	0.001763	0.006263	0.003157
Bosnia and Herzegovina	0.005027	0.018008	0.010531
Belarus	0.001319	0.008114	0.008425
Belize	0.001974	0.015939	0.009044
Bermuda	0.001242	-0.01192	-0.01286
Bolivia (Plurinational State of)	-0.00061	0.016688	0.001988
Brazil	-0.00441	-0.00438	-0.00427
Barbados	0.002374	-0.00077	-0.00058
Brunei Darussalam	-0.00959	-0.00441	-0.00957
Bhutan	0.002149	0.031322	0.009785
Botswana	0.001302	0.00996	0.00641
Central African Republic	0.00126	0.008215	0.006218
Canada	-0.0091	-0.01542	-0.01496
Switzerland	-0.00723	-0.01991	-0.01805
Chile	-0.00407	-0.01216	-0.01089
Cte d'Ivoire	0.002923	0.014109	0.011631
Cameroon	0.0043	0.018784	0.016657
Democratic Republic of the Congo	0.000572	0.010462	0.00859
Congo	0.000886	0.012784	0.010022
Cook Islands	0.004868	0.00958	0.005738
Colombia	-0.00194	-0.01386	-0.01157

Comoros	0.001272	0.005865	0.002298
Cabo Verde	0.001612	0.031044	0.025203
Costa Rica	-0.00196	-0.00715	-0.0062
Cuba	0.000564	0.010747	0.010686
Cyprus	-0.00628	-0.00941	-0.01155
Czechia	0.001413	-0.00039	-0.00048
Germany	-0.00839	0.004225	0.001124
Djibouti	0.002081	0.010735	0.008421
Dominica	0.00134	0.008636	0.007398
Denmark	-0.00844	-0.01891	-0.01895
Dominican Republic	0.001861	0.005976	0.004782
Algeria	0.004949	0.06179	0.005057
Ecuador	0.000968	0.074131	0.012756
Eritrea	0.000936	0.020462	0.012376
Spain	-0.00919	-0.01512	-0.01376
Estonia	0.001851	0.010255	0.003566
Ethiopia	0.000421	0.015672	0.008909
Finland	-0.00838	-0.01026	-0.01114
Fiji	0.003989	0.008646	0.00436
France	-0.00779	-0.00303	-0.00788
Micronesia (Federated States of)	0.004868	0.01679	0.009653
Gabon	0.002335	0.014769	0.012964
United Kingdom	-0.01053	-0.01575	-0.0171
Georgia	0.003662	0.129423	0.04562
Ghana	0.001853	0.013122	0.011258
Guinea	0.003034	0.019593	0.016039
Gambia	0.001567	0.017724	0.013329
Guinea-Bissau	0.00214	0.01438	0.011102
Equatorial Guinea	0.000845	0.027161	0.021062
Greece	-0.0066	0.008423	-0.00208
Grenada	0.002972	0.015464	0.011222
Greenland	-0.00491	-0.02301	-0.02138
Guatemala	0.00403	-0.01372	-0.00197
Guam	0.005087	0.022186	0.010942
Guyana	0.002199	0.015527	0.009629
Honduras	0.00034	0.006273	0.003482
Croatia	-0.00165	-0.00064	-0.00236
Haiti	0.003398	0.003715	0.003429
Hungary	-0.00226	-0.0181	-0.0154
Indonesia	0.002801	0.023075	0.005269
India	0.001878	0.02264	0.007286
Ireland	-0.00924	-0.02628	-0.02533
Iran (Islamic Republic of)	0.004815	0.041707	0.008466
Iraq	0.00515	0.01755	0.004808

Iceland	-0.00728	-0.00713	-0.01044
Israel	-0.00696	-0.00644	-0.0084
Italy	-0.00951	-0.02583	-0.02417
Jamaica	0.00356	0.00247	0.003426
Jordan	0.003146	0.042877	0.002182
Japan	-0.01231	-0.00249	-0.00996
Kazakhstan	0.002505	0.034703	0.010524
Kenya	0.000927	0.01965	0.015404
Kyrgyzstan	-5.9E-05	0.019988	0.011932
Cambodia	0.003376	0.010353	0.002185
Kiribati	0.006364	0.022728	0.011904
Saint Kitts and Nevis	0.001642	0.008081	0.005875
Republic of Korea	-0.01119	-0.00726	-0.01482
Kuwait	0.004377	0.019179	0.00416
Lao People's Democratic Republic	0.000592	0.018478	0.002325
Lebanon	0.008919	0.001563	0.00013
Liberia	0.002311	0.014031	0.010848
Libya	0.007229	0.066116	0.004784
Saint Lucia	0.000229	-0.00825	-0.00735
Sri Lanka	0.003669	0.008161	0.001833
Lesotho	0.001754	0.020068	0.018702
Lithuania	0.000466	0.000889	-0.0002
Luxembourg	-0.00347	-0.01109	-0.01071
Latvia	0.001045	0.004808	0.00236
Morocco	0.004682	0.074385	0.004851
Monaco	-0.00642	0.000199	-0.00401
Republic of Moldova	0.004194	0.008739	0.012905
Madagascar	0.00046	0.00671	0.004759
Maldives	0.001363	0.03217	0.000239
Mexico	-0.00196	-0.0255	-0.0167
Marshall Islands	0.004316	0.024816	0.00944
North Macedonia	0.003208	0.014476	0.006791
Mali	0.002829	0.017762	0.013502
Malta	-0.00683	-0.01038	-0.01163
Myanmar	-0.00334	0.00637	-0.00346
Montenegro	0.003284	0.011529	0.004172
Mongolia	0.002021	0.044197	0.02782
Northern Mariana Islands	0.004601	0.042772	0.015967
Mozambique	0.001857	0.020977	0.018968
Mauritania	0.00136	0.011806	0.008338
Mauritius	0.003498	0.062547	0.028762
Malawi	0.002639	0.019517	0.015985
Malaysia	0.001789	0.015085	0.001753
Namibia	-0.00156	0.010929	0.008494

Niger	0.003287	0.010134	0.006579
Nigeria	0.003681	0.008286	0.006526
Nicaragua	3.88E-05	-0.00026	-0.00302
Niue	0.004738	0.016627	0.007697
Netherlands	-0.01195	-0.00567	-0.00899
Norway	-0.00917	-0.03847	-0.03357
Nepal	0.002002	0.024314	0.007772
Nauru	0.006264	0.009015	0.007152
New Zealand	-0.0111	-0.01355	-0.01694
Oman	0.006863	0.026415	0.005964
Pakistan	0.00217	0.020513	0.007853
Panama	-0.00041	-0.00599	-0.00571
Peru	0.003766	0.009549	0.002418
Philippines	0.00018	0.006435	0.002246
Palau	0.004886	0.016661	0.007423
Papua New Guinea	0.002107	0.017262	0.004678
Poland	-0.00278	0.004553	0.000177
Puerto Rico	0.002444	-0.00035	0.001579
Democratic People's Republic of Korea	0.002244	0.007762	0.000681
Portugal	-0.00583	-0.01471	-0.01387
Paraguay	-0.00048	0.007118	0.002822
Palestine	0.005829	0.004887	0.003109
Qatar	0.005601	0.020357	0.010773
Romania	-1.9E-05	0.010827	0.012963
Russian Federation	0.00384	-0.02418	-0.01893
Rwanda	0.002066	0.002608	-0.0006
Saudi Arabia	0.007447	0.018319	0.004832
Senegal	0.001598	0.018093	0.013144
Singapore	-0.01131	-0.00362	-0.01061
Solomon Islands	0.004577	0.020818	0.008573
Sierra Leone	-0.00015	0.011909	0.008567
El Salvador	0.002924	0.000167	-5.1E-06
San Marino	-0.00645	-0.01696	-0.0139
Somalia	0.002363	-0.00183	-0.00053
Serbia	0.002567	-0.00295	-0.00117
South Sudan	0.003168	0.003794	0.00183
Sao Tome and Principe	0.003086	0.023502	0.020584
Suriname	0.003335	0.002897	0.002271
Slovakia	-0.00078	0.004563	0.001292
Slovenia	9.05E-05	0.009781	0.00523
Sweden	-0.00667	-0.03842	-0.02989
Eswatini	0.001611	0.010082	0.010768
Seychelles	0.004466	0.007656	0.003077

Syrian Arab Republic	0.00392	0.061866	0.00303
Chad	0.001063	0.01787	0.013654
Togo	0.000876	0.013107	0.010345
Thailand	-0.00111	0.018894	-0.00268
Tajikistan	0.000907	0.00124	-0.00082
Tokelau	0.004559	0.014844	0.006698
Turkmenistan	0.000772	0.010673	0.005697
Timor-Leste	0.003309	0.017899	0.004324
Tonga	0.003916	0.010151	0.005831
Trinidad and Tobago	0.000144	-0.00683	-0.00479
Tunisia	0.005771	0.068449	0.006794
Trkiye	0.001542	0.007134	0.001127
Tuvalu	0.004398	0.023784	0.009316
Taiwan (Province of China)	0.000858	0.023795	-0.00073
United Republic of Tanzania	0.003165	0.008967	0.006799
Uganda	0.000997	0.016007	0.012021
Ukraine	0.000643	0.005137	0.004872
Uruguay	-0.00307	0.000864	-0.00436
United States of America	-0.00272	4.23E-05	9.06E-05
Uzbekistan	0.00435	0.038162	0.010525
Saint Vincent and the Grenadines	0.001259	-0.00369	-0.00348
Venezuela (Bolivarian Republic of)	-0.00235	-0.01022	-0.00851
United States Virgin Islands	0.002462	-0.00351	-0.00516
Viet Nam	0.003048	0.019744	0.004659
Vanuatu	0.003997	0.013624	0.006528
Samoa	0.003559	0.012436	0.005391
Yemen	0.0049	0.075873	0.00466
South Africa	-0.00399	0.012349	0.009521
Zambia	0.001257	0.023318	0.021112
Zimbabwe	0.002219	0.007674	0.003366
Egypt	0.00795	0.010819	0.006801
Sudan	0.007202	0.078737	0.006951
China	0.000898	0.007329	-0.00236

LEPAD, lower extremity peripheral arterial disease; ASR, age-standardised rate; ASIR, age-standardised incidence rate; ASMR, age-standardised mortality rate; ASDR, age-standardised disability-adjusted life years; APC, annual percentage change.

Supplementary Table 3 Morbidity and mortality of global and different SDI regions by age group

Location	Measure	Age	Metric	Value(95%UI)
Low SDI	Incidence	35-39 years	Rate	0.00(0.00,0.00)
Global	Incidence	35-39 years	Rate	0.00(0.00,0.00)
Low-middle SDI	Incidence	35-39 years	Rate	0.00(0.00,0.00)
High SDI	Incidence	35-39 years	Rate	0.00(0.00,0.00)
Middle SDI	Incidence	35-39 years	Rate	0.00(0.00,0.00)
High-middle SDI	Incidence	35-39 years	Rate	0.00(0.00,0.00)
Low-middle SDI	Deaths	40-44 years	Rate	0.04(0.03,0.06)
Low SDI	Deaths	40-44 years	Rate	0.06(0.03,0.12)
Global	Deaths	40-44 years	Rate	0.05(0.05,0.07)
Low SDI	Incidence	40-44 years	Rate	46.79(36.81,58.73)
Global	Incidence	40-44 years	Rate	78.49(63.55,96.30)
Low-middle SDI	Incidence	40-44 years	Rate	58.08(46.24,72.68)
Middle SDI	Deaths	40-44 years	Rate	0.05(0.04,0.06)
High-middle SDI	Deaths	40-44 years	Rate	0.06(0.06,0.07)
High SDI	Deaths	40-44 years	Rate	0.07(0.06,0.07)
High SDI	Incidence	40-44 years	Rate	128.17(105.77,151.75)
Middle SDI	Incidence	40-44 years	Rate	73.29(58.36,91.28)
High-middle SDI	Incidence	40-44 years	Rate	90.31(72.88,111.55)
Low-middle SDI	Deaths	45-49 years	Rate	0.06(0.05,0.08)
Low SDI	Deaths	45-49 years	Rate	0.08(0.04,0.15)
Global	Deaths	45-49 years	Rate	0.10(0.09,0.11)
Low SDI	Incidence	45-49 years	Rate	76.24(49.07,108.82)
Global	Incidence	45-49 years	Rate	123.15(80.98,172.65)
Low-middle SDI	Incidence	45-49 years	Rate	95.33(61.52,136.17)
Middle SDI	Deaths	45-49 years	Rate	0.08(0.07,0.09)
High-middle SDI	Deaths	45-49 years	Rate	0.13(0.12,0.15)
High SDI	Deaths	45-49 years	Rate	0.15(0.14,0.16)
High SDI	Incidence	45-49 years	Rate	146.15(98.99,203.45)
Middle SDI	Incidence	45-49 years	Rate	128.06(82.90,179.95)
High-middle SDI	Incidence	45-49 years	Rate	146.02(95.07,205.22)
Low-middle SDI	Deaths	50-54 years	Rate	0.16(0.12,0.22)
Low SDI	Deaths	50-54 years	Rate	0.27(0.15,0.54)
Global	Deaths	50-54 years	Rate	0.23(0.21,0.28)
Low SDI	Incidence	50-54 years	Rate	124.12(92.62,164.18)
Global	Incidence	50-54 years	Rate	191.34(143.42,250.38)
Low-middle SDI	Incidence	50-54 years	Rate	152.41(113.70,199.99)
Middle SDI	Deaths	50-54 years	Rate	0.15(0.13,0.17)
High-middle SDI	Deaths	50-54 years	Rate	0.30(0.27,0.35)
High SDI	Deaths	50-54 years	Rate	0.40(0.38,0.43)
High SDI	Incidence	50-54 years	Rate	213.75(158.28,284.58)

Middle SDI	Incidence	50-54 years	Rate	198.33(148.28,259.13)
High-middle SDI	Incidence	50-54 years	Rate	219.32(164.52,285.47)
Low-middle SDI	Deaths	50-69 years	Rate	0.46(0.36,0.61)
Low SDI	Deaths	50-69 years	Rate	0.83(0.44,1.55)
Global	Deaths	50-69 years	Rate	0.85(0.77,0.96)
Low SDI	Incidence	50-69 years	Rate	224.56(177.61,286.49)
Global	Incidence	50-69 years	Rate	352.82(279.35,447.53)
Low-middle SDI	Incidence	50-69 years	Rate	272.28(214.55,347.20)
Middle SDI	Deaths	50-69 years	Rate	0.41(0.37,0.46)
High-middle SDI	Deaths	50-69 years	Rate	1.26(1.15,1.38)
High SDI	Deaths	50-69 years	Rate	1.49(1.41,1.58)
High SDI	Incidence	50-69 years	Rate	500.50(395.89,625.55)
Middle SDI	Incidence	50-69 years	Rate	322.76(255.07,410.78)
High-middle SDI	Incidence	50-69 years	Rate	372.25(293.11,476.40)
Low-middle SDI	Deaths	50-74 years	Rate	0.62(0.49,0.83)
Low SDI	Deaths	50-74 years	Rate	1.10(0.60,2.07)
Global	Deaths	50-74 years	Rate	1.19(1.08,1.35)
Low SDI	Incidence	50-74 years	Rate	254.23(206.09,316.66)
Global	Incidence	50-74 years	Rate	405.61(329.90,500.76)
Low-middle SDI	Incidence	50-74 years	Rate	306.90(248.49,382.45)
Middle SDI	Deaths	50-74 years	Rate	0.55(0.49,0.61)
High-middle SDI	Deaths	50-74 years	Rate	1.72(1.57,1.91)
High SDI	Deaths	50-74 years	Rate	2.17(2.04,2.31)
High SDI	Incidence	50-74 years	Rate	601.35(493.49,731.25)
Middle SDI	Incidence	50-74 years	Rate	357.82(290.93,445.68)
High-middle SDI	Incidence	50-74 years	Rate	420.67(338.30,522.15)
Low-middle SDI	Deaths	75-79 years	Rate	3.68(2.87,4.77)
Low SDI	Deaths	75-79 years	Rate	7.11(3.83,13.10)
Global	Deaths	75-79 years	Rate	6.28(5.71,6.92)
Low SDI	Incidence	75-79 years	Rate	596.38(398.76,842.16)
Global	Incidence	75-79 years	Rate	831.29(562.11,1162.93)
Low-middle SDI	Incidence	75-79 years	Rate	657.54(438.16,922.71)
Middle SDI	Deaths	75-79 years	Rate	2.88(2.59,3.17)
High-middle SDI	Deaths	75-79 years	Rate	7.24(6.67,7.78)
High SDI	Deaths	75-79 years	Rate	10.41(9.49,11.04)
High SDI	Incidence	75-79 years	Rate	1135.66(785.11,1560.93)
Middle SDI	Incidence	75-79 years	Rate	692.70(463.81,977.39)
High-middle SDI	Incidence	75-79 years	Rate	811.56(545.14,1143.48)
Low-middle SDI	Deaths	80-84 years	Rate	6.60(5.06,8.64)
Low SDI	Deaths	80-84 years	Rate	12.48(6.78,23.40)
Global	Deaths	80-84 years	Rate	13.21(11.67,14.33)
Low SDI	Incidence	80-84 years	Rate	643.30(451.77,871.77)
Global	Incidence	80-84 years	Rate	849.03(597.57,1164.51)
Low-middle SDI	Incidence	80-84 years	Rate	693.57(483.84,945.38)

Middle SDI	Deaths	80-84 years	Rate	5.45(4.79,6.00)
High-middle SDI	Deaths	80-84 years	Rate	17.57(15.48,19.19)
High SDI	Deaths	80-84 years	Rate	19.39(16.44,21.13)
High SDI	Incidence	80-84 years	Rate	1071.90(746.08,1461.04)
Middle SDI	Incidence	80-84 years	Rate	716.74(501.17,984.12)
High-middle SDI	Incidence	80-84 years	Rate	831.90(579.70,1149.15)
Low-middle SDI	Deaths	85-89 years	Rate	11.53(8.44,15.08)
Low SDI	Deaths	85-89 years	Rate	21.30(10.88,41.23)
Global	Deaths	85-89 years	Rate	26.66(22.38,29.50)
Low SDI	Incidence	85-89 years	Rate	658.61(419.50,912.98)
Global	Incidence	85-89 years	Rate	820.16(536.56,1136.47)
Low-middle SDI	Incidence	85-89 years	Rate	691.85(439.21,967.38)
Middle SDI	Deaths	85-89 years	Rate	10.26(8.78,11.32)
High-middle SDI	Deaths	85-89 years	Rate	32.32(27.52,36.33)
High SDI	Deaths	85-89 years	Rate	39.09(32.03,43.34)
High SDI	Incidence	85-89 years	Rate	969.50(647.06,1321.46)
Middle SDI	Incidence	85-89 years	Rate	708.33(454.47,993.33)
High-middle SDI	Incidence	85-89 years	Rate	791.91(519.64,1104.82)
Low-middle SDI	Deaths	90-94 years	Rate	20.81(14.65,27.54)
Low SDI	Deaths	90-94 years	Rate	37.49(19.50,70.21)
Global	Deaths	90-94 years	Rate	55.47(43.81,61.59)
Low SDI	Incidence	90-94 years	Rate	687.71(445.16,948.79)
Global	Incidence	90-94 years	Rate	804.00(507.97,1134.58)
Low-middle SDI	Incidence	90-94 years	Rate	711.82(463.45,990.84)
Middle SDI	Deaths	90-94 years	Rate	20.09(16.35,22.47)
High-middle SDI	Deaths	90-94 years	Rate	61.90(49.73,68.32)
High SDI	Deaths	90-94 years	Rate	77.61(59.89,86.63)
High SDI	Incidence	90-94 years	Rate	886.52(556.26,1241.93)
Middle SDI	Incidence	90-94 years	Rate	725.07(470.12,1019.33)
High-middle SDI	Incidence	90-94 years	Rate	772.82(488.97,1100.63)
Low-middle SDI	Deaths	95+ years	Rate	32.45(21.74,43.73)
Low SDI	Deaths	95+ years	Rate	47.20(23.11,90.37)
Global	Deaths	95+ years	Rate	101.61(73.98,115.79)
Low SDI	Incidence	95+ years	Rate	732.30(419.27,1179.15)
Global	Incidence	95+ years	Rate	780.94(456.55,1256.28)
Low-middle SDI	Incidence	95+ years	Rate	759.23(433.54,1215.17)
Middle SDI	Deaths	95+ years	Rate	36.39(27.57,42.05)
High-middle SDI	Deaths	95+ years	Rate	107.65(81.13,121.83)
High SDI	Deaths	95+ years	Rate	136.32(97.74,156.11)
High SDI	Incidence	95+ years	Rate	800.60(475.60,1295.17)
High-middle SDI	Incidence	95+ years	Rate	764.45(442.05,1236.86)
Middle SDI	Incidence	95+ years	Rate	759.36(433.82,1225.93)

SDI, social demographic index; UI, uncertainty interval.

Supplementary Table 4 The AAPC of global LEPAD ASIR and ASMR from 1990 to 2021

Metric	sex_name	Start	End	AAPC	AAPC CI. Low	AAPC CI. High	Test Statistic	P-Value
ASIR	Both	1990	2021	-0.40	-0.42	-0.38	-37.55	0
ASIR	Female	1990	2021	-0.38	-0.42	-0.34	-19.27	0
ASIR	Male	1990	2021	-0.37	-0.39	-0.35	-32.59	0
ASMR	Both	1990	2021	-1.46	-1.94	-0.98	-5.90	0
ASMR	Female	1990	2021	-1.45	-1.74	-1.15	-9.47	0
ASMR	Male	1990	2021	-1.44	-1.83	-1.05	-7.13	0

LEPAD, lower extremity peripheral arterial disease; ASIR, age-standardised incidence rate; ASMR, age-standardised mortality rate; AAPC, average annual percentage change; CI, confidence interval.

Supplementary Table 5 APC of global LEPAD ASIR and ASMR from 1990 to 2021

Metric	sex_name	Segment Start	Segment End	APC	APC 95% LCL	APC 95% UCL	Test Statistic	P-Value
ASIR	Both	1990	2007	-0.50	-0.52	-0.49	-68.35	0.00
ASIR	Both	2007	2015	-0.39	-0.45	-0.34	-14.33	0.00
ASIR	Both	2015	2021	-0.11	-0.18	-0.04	-3.17	0.00
ASIR	Female	1990	2000	-0.41	-0.45	-0.37	-21.63	0.00
ASIR	Female	2000	2004	-0.61	-0.87	-0.36	-5.07	0.00
ASIR	Female	2004	2014	-0.42	-0.46	-0.37	-18.80	0.00
ASIR	Female	2014	2021	-0.15	-0.22	-0.08	-4.52	0.00
ASIR	Male	1990	1996	-0.62	-0.67	-0.57	-25.90	0.00
ASIR	Male	1996	2008	-0.44	-0.46	-0.42	-47.16	0.00
ASIR	Male	2008	2019	-0.28	-0.30	-0.25	-26.59	0.00
ASIR	Male	2019	2021	0.31	0.02	0.60	2.20	0.04
ASMR	Both	1990	1994	1.03	0.04	2.03	2.22	0.04
ASMR	Both	1994	1997	-1.50	-4.26	1.34	-1.13	0.28
ASMR	Both	1997	2000	0.48	-2.32	3.37	0.36	0.72
ASMR	Both	2000	2007	-2.70	-3.20	-2.20	-11.34	0.00
ASMR	Both	2007	2010	-0.86	-3.96	2.33	-0.58	0.57
ASMR	Both	2010	2021	-2.23	-2.46	-1.99	-20.28	0.00
ASMR	Female	1990	1993	0.87	-0.53	2.29	1.33	0.20
ASMR	Female	1993	2000	-0.23	-0.65	0.19	-1.17	0.26
ASMR	Female	2000	2006	-2.67	-3.25	-2.09	-9.69	0.00
ASMR	Female	2006	2013	-1.44	-1.91	-0.96	-6.35	0.00
ASMR	Female	2013	2017	-3.54	-4.97	-2.09	-5.13	0.00
ASMR	Female	2017	2021	-1.34	-2.35	-0.32	-2.79	0.01
ASMR	Male	1990	1994	0.99	0.17	1.83	2.57	0.02
ASMR	Male	1994	1997	-2.24	-4.51	0.08	-2.06	0.06
ASMR	Male	1997	2000	0.63	-1.67	2.99	0.58	0.57
ASMR	Male	2000	2007	-2.73	-3.13	-2.33	-14.45	0.00
ASMR	Male	2007	2010	-1.09	-3.58	1.47	-0.91	0.38
ASMR	Male	2010	2021	-1.92	-2.13	-1.72	-19.76	0.00

LEPAD, lower extremity peripheral arterial disease; ASIR, age-standardised incidence rate; ASMR, age-standardised mortality rate; APC, annual percentage change; LCL, lower control limit; UCL, upper control limit.

Supplementary Table 6 Regional differences in SEV of metabolic risk factors in 2021

Location	High LDL-C (SEV%)	High blood pressure (SEV%)	High body-mass index (SEV%)	Dominant risk
Global	45.3	35.63	21.49	High LDL
Low SDI	33.03	35.78	12.46	High blood pressure
Low-middle SDI	37.7	36.8	16.9	High LDL
Middle SDI	45.61	35.41	21.6	High LDL
High-middle SDI	51.35	37.97	25.43	High LDL
High SDI	53.69	31.33	32.55	High LDL

SEV, summary exposure value; SDI, social demographic index; LDL-C, Low-Density Lipoprotein Cholesterol.

Supplementary Table 7 Global incidence of LEPAD by gender, 1990–2050: based on the BAPC prediction model

Both		Male		Female	
number	year	number	year	number	year
1.08294E+16	1990	1.08294E+16	1990	1102168071	1990
1.0746E+16	1991	1.0746E+16	1991	1089299798	1991
1.06776E+16	1992	1.06776E+16	1992	1074972406	1992
1.06425E+16	1993	1.06425E+16	1993	1064863813	1993
1.06536E+16	1994	1.06536E+16	1994	1061323232	1994
1.07213E+16	1995	1.07213E+16	1995	1063419146	1995
1.08306E+16	1996	1.08306E+16	1996	1067758153	1996
1.09705E+16	1997	1.09705E+16	1997	1074238208	1997
1.11415E+16	1998	1.11415E+16	1998	1083411445	1998
1.13371E+16	1999	1.13371E+16	1999	1095253876	1999
1.1554E+16	2000	1.1554E+16	2000	1109437774	2000
1.17955E+16	2001	1.17955E+16	2001	1125188260	2001
1.20377E+16	2002	1.20377E+16	2002	1140248008	2002
1.22521E+16	2003	1.22521E+16	2003	1152453442	2003
1.24179E+16	2004	1.24179E+16	2004	1160442970	2004
1.25178E+16	2005	1.25178E+16	2005	1162859102	2005
1.25335E+16	2006	1.25335E+16	2006	1156988832	2006
1.24871E+16	2007	1.24871E+16	2007	1145196482	2007
1.24048E+16	2008	1.24048E+16	2008	1130676591	2008
1.23042E+16	2009	1.23042E+16	2009	1115971619	2009
1.21944E+16	2010	1.21944E+16	2010	1102622546	2010
1.21048E+16	2011	1.21048E+16	2011	1091643291	2011
1.20399E+16	2012	1.20399E+16	2012	1083621773	2012
1.19906E+16	2013	1.19906E+16	2013	1078327493	2013
1.19561E+16	2014	1.19561E+16	2014	1076109102	2014
1.19452E+16	2015	1.19452E+16	2015	1078034860	2015
1.19539E+16	2016	1.19539E+16	2016	1083278844	2016
1.19823E+16	2017	1.19823E+16	2017	1092348396	2017
1.20345E+16	2018	1.20345E+16	2018	1104610964	2018
1.21143E+16	2019	1.21143E+16	2019	1118291976	2019
1.22296E+16	2020	1.22296E+16	2020	1132896034	2020
1.24047E+16	2021	1.24047E+16	2021	1161408345	2021
1.27203E+16	2022	1.27203E+16	2022	1191710399	2022
1.28606E+16	2023	1.28606E+16	2023	1219080430	2023
1.30057E+16	2024	1.30057E+16	2024	1249683389	2024
1.31519E+16	2025	1.31519E+16	2025	1283064934	2025

1.32949E+16	2026	1.32949E+16	2026	1318748181	2026
1.34305E+16	2027	1.34305E+16	2027	1356205965	2027
1.35578E+16	2028	1.35578E+16	2028	1395707989	2028
1.3678E+16	2029	1.3678E+16	2029	1437827685	2029
1.37895E+16	2030	1.37895E+16	2030	1482405823	2030
1.38905E+16	2031	1.38905E+16	2031	1529245341	2031
1.39958E+16	2032	1.39958E+16	2032	1579939495	2032
1.40394E+16	2033	1.40394E+16	2033	1628508178	2033
1.40668E+16	2034	1.40668E+16	2034	1679712808	2034
1.40838E+16	2035	1.40838E+16	2035	1734752388	2035
1.40942E+16	2036	1.40942E+16	2036	1794738063	2036
1.4088E+16	2037	1.4088E+16	2037	1859120285	2037
1.41273E+16	2038	1.41273E+16	2038	1935115407	2038
1.41581E+16	2039	1.41581E+16	2039	2017085870	2039
1.41787E+16	2040	1.41787E+16	2040	2105441481	2040
1.41919E+16	2041	1.41919E+16	2041	2201290636	2041
1.41996E+16	2042	1.41996E+16	2042	2305763718	2042
1.42038E+16	2043	1.42038E+16	2043	2420150220	2043
1.42079E+16	2044	1.42079E+16	2044	2546172910	2044
1.42149E+16	2045	1.42149E+16	2045	2685715334	2045
1.42261E+16	2046	1.42261E+16	2046	2840720720	2046
1.42411E+16	2047	1.42411E+16	2047	3013037120	2047
1.42594E+16	2048	1.42594E+16	2048	3204723587	2048
1.42815E+16	2049	1.42815E+16	2049	3418471784	2049
1.43084E+16	2050	1.43084E+16	2050	3657478304	2050

LEPAD, lower extremity peripheral arterial disease; BAPC, Bayesian age-period-cohort models.
