# Supplementary figures and images for: Global, regional, and national burdens of lower extremity peripheral arterial disease from 1990 to 2021 and projections to 2050: global burden of disease study 2021
Source: Front Cardiovasc Med. 2025 Oct 7;12:1592322. doi: 10.3389/fcvm.2025.1592322 (PMC12537751; doi:10.3389/fcvm.2025.1592322)

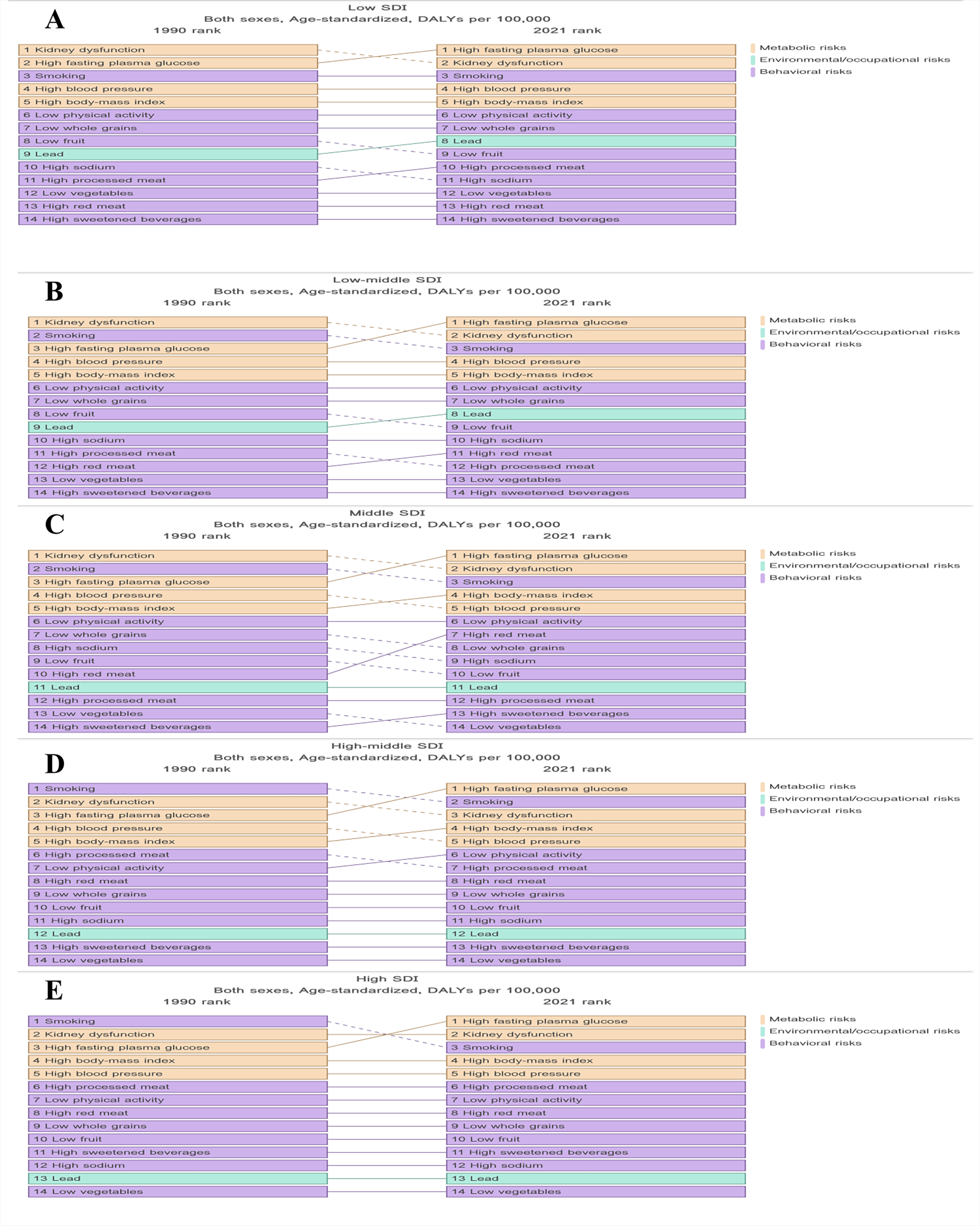

Supplement: Supplementary file 2 [file Image1.tif]
